# Carboxylate-assisted C–H activation of phenylpyridines with copper, palladium and ruthenium: a mass spectrometry and DFT study†
†Electronic supplementary information (ESI) available: Details on the mass-spectrometry experiments and theoretical calculations, Hammett studies, potential energy surfaces, energies, optimized Gaussian geometries and laser-power dependence during the IRMPD spectra measurements. See DOI: 10.1039/c5sc01729g


**DOI:** 10.1039/c5sc01729g

**Published:** 2015-07-01

**Authors:** A. Gray, A. Tsybizova, J. Roithova

**Affiliations:** a Department of Organic Chemistry , Faculty of Science , Charles University in Prague , Hlavova 2030/8 , 12843 Prague 2 , Czech Republic . Email: roithova@natur.cuni.cz

## Abstract

The C–H activation of 2-phenylpyridine, catalyzed by copper(ii), palladium(ii) and ruthenium(ii) carboxylates, was studied in the gas phase. ESI-MS, infrared multiphoton dissociation spectroscopy and quantum chemical calculations were combined to investigate the intermediate species in the reaction. Collision induced dissociation (CID) experiments and DFT calculations allowed estimation of the energy required for this C–H activation step and the subsequent acetic acid loss. Hammett plots constructed from the CID experiments using different copper carboxylates as catalysts revealed that the use of stronger acids accelerates the C–H activation step. The reasoning can be traced from the associated transition structures that suggest a concerted mechanism and the key effect of the carbon–metal bond pre-formation. Carboxylates derived from stronger acids make the metal atom more electrophilic and therefore shift the reaction towards the formation of C–H activated products.

## Abstract 2

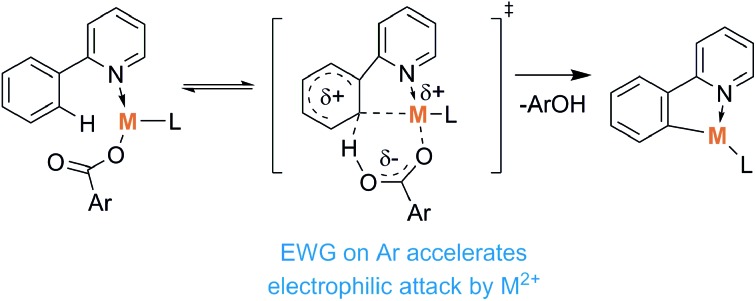
The transition state of metal carboxylate mediated C–H activation is associated with carbon–metal bond formation supported by electron-poor carboxylates.

## Introduction

C–H bond activation is an important topic in modern organic synthesis.^1^–^3^ While C–H activation in pure hydrocarbons is still a challenge,^4^–^6^ there are many approaches to activate the C–H bonds of functionalized molecules. Most methods involve transition-metal catalysis where a continuum of reaction mechanisms exists. Within this continuum examples of traditional limiting cases are (i) oxidative addition with electron-rich late transition metals, (ii) σ-bond metathesis with early transition metals, and (iii) electrophilic activation with electron-deficient late transition metals.^7^–^26^

**Scheme 1:**
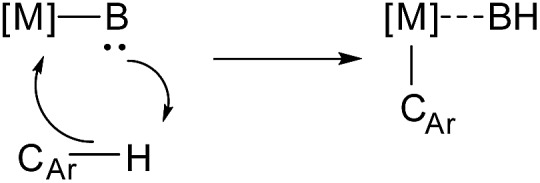
Metal/base promoted C–H bond activation.

Some C–H activation reactions were shown to proceed much more efficiently and selectively through so-called ligand direction.^27^–^38^ Such assistance is well known for metal catalysts with ligands acting as bases (base-assisted C–H activation, Scheme 1).^31^

One of the first examples of such a reaction was reported in 1955 by Winstein and Traylor for the acetolysis of diphenylmercury in acetic acid.^39^ Early mechanistic studies proposed an electrophilic aromatic substitution mechanism,^40^–^43^ for which large primary kinetic isotope effects were observed.^44^ Therefore proton transfer was suggested to be the rate limiting step. However, recent mechanistic investigations have shown that C–H bond cleavage can proceed *via* a simultaneous metalation and intramolecular deprotonation: the so-called concerted metalation-deprotonation (CMD) mechanism.^31^,^45^–^47^

Among many variations of base-assisted C–H activation, carboxylate-assisted C–H activation is one of the most widely used in synthesis, generally considered to require mild reaction conditions.^26^,^31^ Although these reactions are usually performed with unsaturated heterocycles or hydrocarbons,^11^,^26^,^47^–^55^ a few examples of sp^3^ C–H bond activation have also been reported.^26^,^56^,^57^ In general, the reactions proceed *via* a metallocyclic structure, however different pathways have been proposed depending on the origin of the metal and the structure of the substrate.^26^,^47^,^49^,^56^–^66^

**Scheme 2:**
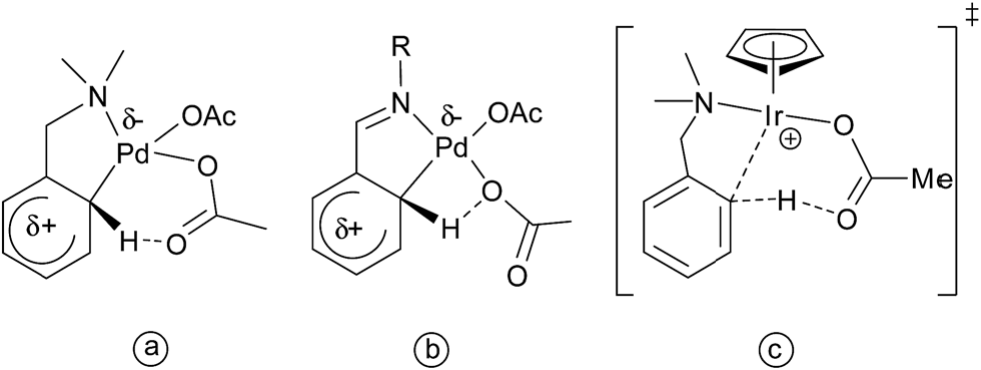
Suggested transition states for the palladium acetate assisted C–H activation of a substituted (a) amine and (b) imine and (c) the transition state for the iridium catalyzed activation.

For example, in the case of palladium acetate and *N*,*N*-dimethylbenzylamine, Ryabov and co-workers proposed an electrophilic mechanism for the transfer of a proton to acetate through a highly ordered six-membered transition state (Scheme 2a).^67^,^68^ Subsequent studies on imine cyclometalation reactions have suggested a four-membered transition state (Scheme 2b).^69^ For iridium complexes, computational studies by Davies, Macgregor, and co-workers, suggested the formation of a transition state shown at the Scheme 2c.^70^–^72^ The possibility of an oxidative addition pathway with or without acetate assistance has also been considered.^73^ Recently Flegeau and co-workers have suggested that Ru-catalyzed activation of 2-phenylpyridine (2-PhPy) might go *via* a S_E_3 mechanism, based on their NMR kinetic studies.^74^

**Scheme 3:**
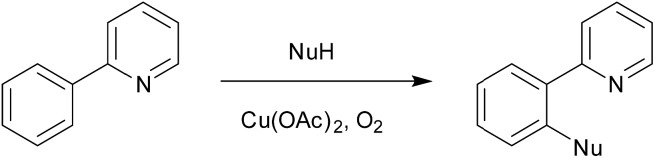
Cu(ii)-catalyzed activation of aryl C–H bonds.

Copper acetate was also found to be capable of activating 2-phenylpyridine, enabling subsequent substitution by nucleophiles (Scheme 3).^75^,^76^

**Scheme 4:**
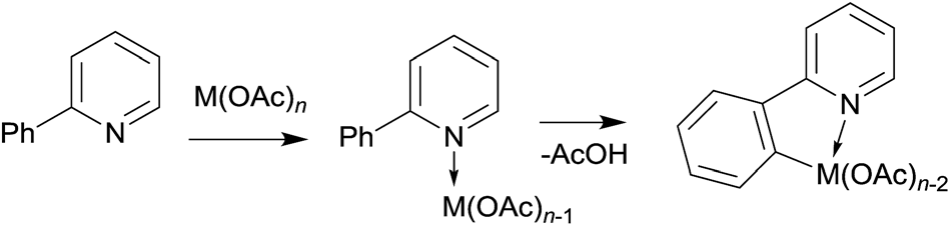
The carboxylate assisted C–H activation investigated here. M was Ru, Cu, and Pd.

We have decided to investigate the behavior of 2-phenylpyridine with three different metal carboxylates (ruthenium, palladium and copper) by means of ESI-MS, infrared multiphoton dissociation spectroscopy (IRMPD) and DFT calculations (Scheme 4) in order to compare their modes of activation and structures of the reaction intermediates.

## Results

### ESI-MS and CID studies

**Fig. 1:**
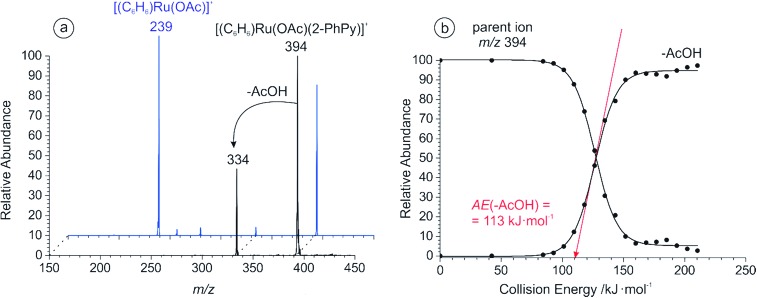
(a) CID spectra of the mass selected peak at *m*/*z* 394 for 2-PhPy (black) and 3-PhPy (blue); (b) the breakdown curve for *m*/*z* 394 (2-PhPy). The appearance energy for acetic acid loss was determined to be AE(–AcOH) = 113 ± 3 kJ mol^–1^.

In the case of ruthenium carboxylate catalyzed C–H activation, the reactive catalytic species had to be generated *in situ* from the dimeric complex: [(C_6_H_6_)RuCl_2_]_2_. This required the presence of a carboxylic acid and a base in the reaction mixture.^77^ We therefore prepared a 0.1 mM solution of the corresponding ruthenium dimer in acetonitrile, and added 1 equivalent of acetic acid and triethylamine. Triethylamine is soluble in acetonitrile and suitable for ESI-MS experiments with the resulting spectrum shown in Fig. S1 of the ESI.† Following the addition of 1 equivalent of 2-phenylpyridine to the corresponding solution, ESI-MS revealed the formation of an ion at *m*/*z* 394 that contained both catalytic and substrate parts (Fig. S2a†). CID analysis of this species showed the elimination of a neutral acetic acid fragment leading to the [(C_6_H_6_)Ru((2-PhPy)-H)]^+^ complex containing C–H activated 2-phenylpyridine (Fig. 1). Fragmentation of the parent ions (*m*/*z* 394) can either represent elimination of neutral acetic acid from the activated complex (*i.e.* [(C_6_H_6_)Ru((2-PhPy)-H)(AcOH)]^+^) or the result of C–H activation in the non-activated complex (*i.e.* [(C_6_H_6_)Ru(2-PhPy)(AcO)]^+^). We will show that the latter option is correct. Increasing the concentration of 2-phenylpyridine was shown to increase the abundance of both non-activated and activated (*m*/*z* 394 and *m*/*z* 334) ruthenium complexes (see Fig. S4 in the ESI†).

We have compared the ability of ruthenium carboxylate to activate 3-phenylpyridine under the same reaction conditions. The ESI mass spectrum of the equivalent solution with 3-phenylpyridine is shown in Fig. S5 of the ESI.† CID analysis of the ion at *m*/*z* 394 showed that although the C–H activation pathway is still present, the elimination of a neutral 3-phenylpyridine molecule is more favorable (Fig. 1a, blue), and the energy required to eliminate acetic acid is much larger than with 2-phenylpyridine: 173 ± 3 kJ mol^–1^ (see Fig. S6 at the ESI†).

The ESI mass spectrum of copper acetate and 2-phenylpyridine (1 : 1) in acetonitrile is shown in Fig. S2b.† All of the complexes containing a 2-phenylpyridine moiety and acetate were subjected to CID to test if C–H activation would occur. The complex at *m*/*z* 432 formally containing Cu(OAc)^+^ and two 2-PhPy units showed competition between the elimination of 2-phenylpyridine and acetic acid (Fig. S7 at the ESI†). The determined appearance energies stood at 147 ± 5 kJ mol^–1^ for 2-phenylpyridine loss and to 138 ± 5 kJ mol^–1^ for acetic acid loss. While the former fragmentation is more abundant, its appearance energy is higher than for the latter fragmentation.

These observations suggest that the elimination of 2-phenylpyridine is kinetically favored and that the elimination of acetic acid represents the C–H activation step proceeding *via* a tight transition state rather than just a facile ligand loss. Therefore the structure of the complex most probably corresponds to non-activated [Cu(OAc)(2-PhPy)_2_]^+^.

The mass spectrum obtained from a mixture of Pd(OAc)_2_ and 2-phenylpyridine in acetonitrile is shown in Fig. S2c.† We have observed complexes with a Pd–C bond (*m*/*z* 415 and *m*/*z* 570) together with possibly non-activated complexes containing 2 and 3 molecules of 2-phenylpyridine at *m*/*z* 475, and 630 respectively. However, they are present in small abundances, as most of the 2-phenylpyridine is already activated (complexes at *m*/*z* 415, 456, 570 *etc.*). In parallel with the copper complexes above reported, the structure of the parent ion at *m*/*z* 475 may correspond to [Pd(OAc)(2-PhPy)_2_]^+^.

CID of this complex exclusively leads to elimination of acetic acid. Our estimated value for the acetic acid loss is 65 ± 3 kJ mol^–1^ (Fig. S8†) (note that the experiment was performed with the isolation width of 10 *m*/*z* with the calibration done specifically for that case). As the AE for elimination of acetic acid is much lower than in the previous complexes, it may be possible that acetic acid is bound as a neutral ligand and the ions at *m*/*z* 475 represent complexes with activated 2-phenylpyridine [Pd((2-PhPy)-H)(2-PhPy)(AcOH)]^+^. CID analysis of the complexes containing more 2-phenylpyridine ligands (*e.g.* the complex at *m*/*z* 630) showed the elimination of neutral 2-phenylpyridine and subsequent C–H activation (see Fig. S9 in the ESI†).

### Reaction pathways: a theoretical study

**Fig. 2:**
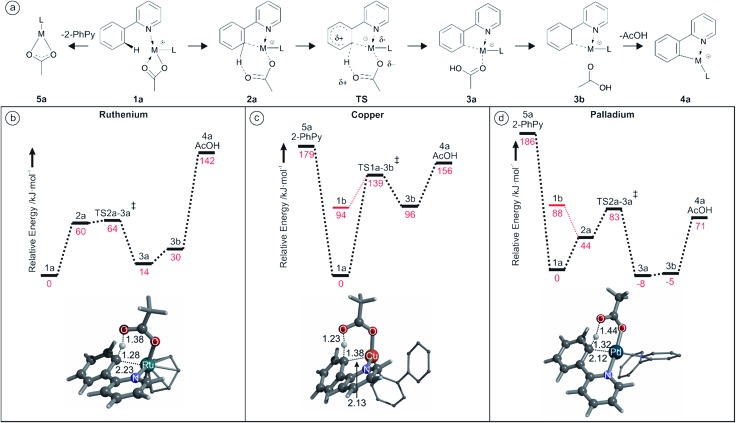
(a) Structural representations of the different calculated species involved in the C–H activation reactions (structure 1b the monodentate isomer, was omitted for clarity); (b) potential energy surface (B3LYP/cc-pVTZ:cc-pVTZ-pp(Ru)) for the Ru assisted C–H activation of 2-PhPy.; (c) potential energy surface (B3LYP/cc-pVTZ:cc-pVTZ-pp(Cu)) for the Cu assisted C–H activation of 2-PhPy.; (d) potential energy surface (B3LYP/cc-pVTZ:cc-pVTZ-pp(Pd)) for the Pd assisted C–H activation of 2-PhPy. All energies are at 0 K and the depicted structures represent the transition structures; distances are in Å.

DFT calculations were used to study the acetate assisted C–H activation of 2-PhPy with Ru, Cu and Pd catalysts. Each of the derived mechanisms exhibited several common steps and similar labelling will be employed to aid their comparison. An overall guide to the progression of these reactions is shown in Fig. 2a (structure 1b representing the non-activated complex bearing monodentate acetate was omitted for clarity).

However, not all of the steps were present for each catalyst. In the Ru examples the generic ligand (L) represents a π-bonded benzene, while with Cu or Pd L is the supporting 2-PhPy bonded to the metal through the nitrogen atom.

Labelling is consistent for all metals with the metal indicated in this text using superscript prefixes as follows: ^Ru^1_a_, ^Cu^1_a_ or ^Pd^1_a_.

Fig. 2b shows the calculated reaction pathway for [(2-PhPy)Ru(C_6_H_6_)(OAc)]^+^. As was previously proposed, the calculated mechanism includes proton transfer from the 2-PhPy ligand directly to the acetate ligand. The reaction begins with the low energy intermediate ^Ru^1_a_ where Ru is bound to the acetate through both oxygen atoms. In the next step (^Ru^2_a_) the acetate is bound through a single oxygen atom while the other is weakly coordinated to 2-PhPy through the proton at the activation site. This structure lies 60 kJ mol^–1^ higher in energy than ^Ru^1_a_ and will be referred to as pre-activated as it allows for easy proton transfer. It additionally features a weak interaction between the Ru and the carbon at the activation site of 2-PhPy.

The activation energy for hydrogen transfer from the pre-activated structure is only 4 kJ mol^–1^. The transition state (^Ru^TS_2a–3a_) leads directly to the activated intermediate ^Ru^3_a_ where acetic acid has been formed. The C–H activation process within the complex is endothermic (14 kJ mol^–1^). The ^Ru^3_a_ complex is stabilized by a weak interaction between the OH group and 2-PhPy. Removal of this stabilization costs 16 kJ mol^–1^ and results in the ^Ru^3_b_ intermediate with acetic acid only bound to the metal ion. Elimination of acetic acid takes place with an additional energy cost of 112 kJ mol^–1^ giving an overall energy increase for this transformation of 142 kJ mol^–1^.

The calculated potential energy surface for Cu activation seen in Fig. 2c has some notable differences from Ru however the steps follow a similar progression. Despite the fact that the supporting ligand has been changed from benzene to 2-PhPy the reaction still proceeds through structures ^Cu^1_a_, ^Cu^TS_1a–3b_, ^Cu^3_b_ and ^Cu^4_a_. However, the energy difference for each of the steps is vastly different when compared with Fig. 2b. An additional initial structure (^Cu^1_b_) is shown in Fig. 2c. ^Cu^1_b_ has a single oxygen atom of the acetate ligand coordinating to the metal center. This has a large (94 kJ mol^–1^) energy penalty and is significantly disfavored. The activation energy for hydrogen transfer from 2-PhPy to acetate is now 139 kJ mol^–1^ which is considerably greater than the 64 kJ mol^–1^ for Ru. In the transition structure the Ru to H distance is 2.22 Å while the same Cu to H distance was 2.24 Å. When the relative size of the two metal ions is taken into account (second row *vs.* first row) the observed consistency in the M–H distances is surprising. This indicates that the larger Ru metal will have a greater influence on the transition structure and probably support the hydrogen transfer resulting in the lower activation barrier. Another considerable difference is found for the endothermicity of C–H activation measured by the energy difference between the activated and non-activated complexes (*i.e.* 3_b_ and 1_a_). With Cu this difference is 96 kJ mol^–1^ while with Ru it was only 30 kJ mol^–1^. Elimination of acetic acid from ^Cu^3_b_ then only requires 60 kJ mol^–1^ which corresponds to an overall energy demand of 156 kJ mol^–1^.

Fig. 2d shows that the calculated reaction pathway for Pd activation was again different from the previous examples. Firstly, the C–H activation within the complex is exothermic. Secondly, the highest point on the potential energy surface corresponds to the transition structure (^Pd^TS_2a–3a_) for C–H activation leading to the final acetic-acid elimination.

A comparison is once again made between the monodentate and bidentate versions of the starting conformation. In this case, the monodentate isomer is 88 kJ mol^–1^ less stable that the bidentate example which is close to the 94 kJ mol^–1^ difference for copper. This indicates that changing the metal does not appear to have a large impact at this stage. Then, similar to the Ru but different from the Cu case, a distorted bidentate isomer (^Pd^2_a_) was located. This is arranged in such a way as to bring the C–H activation site of the 2-PhPy ligand into the vicinity of the metal. The proximity of the metal would seem significant, with short distances present (Pd–O 3.00 Å, Pd–H 2.67 Å and Pd–C 3.14 Å). However, the equivalent Ru structure appeared to have a stronger metal to carbon association (Ru–O 3.27 Å, Ru–H 2.34 Å and Ru–C 2.48 Å).

The transition structure was also similar to those observed with Ru and Cu with an activation energy of 83 kJ mol^–1^. That is higher than the equivalent value for Ru (64 kJ mol^–1^) but lower than the Cu value (139 kJ mol^–1^). Meanwhile the Pd–H distance in the transition structure was the smallest (1.97 Å) of the three metals indicating a strong metal influence. Possibly the most significant difference with the Pd is the relative energy of the activated structures with the acetic acid still bound (^Pd^3_a/b_). These structures are actually more stable than the given starting structures rendering the C–H activation step exothermic. The high stability of these structures may originate in their ability to adopt square planar conformations. The equivalent structure for Cu (^Cu^3_b_) was close to tetrahedral and very unstable compared to ^Cu^1_a_. The theoretical energy required for elimination of AcOH from ^Pd^3_a_ (79 kJ mol^–1^) is lower than the proton transfer barrier from ^Pd^1_a_ (83 kJ mol^–1^).

### IRMPD studies

IRMPD experiments were performed on the aforementioned [(2-PhPy)Ru(C_6_H_6_)(OAc)]^+^, [(2-PhPy)_2_Cu(OAc)]^+^ and [(2-PhPy)_2_Pd(OAc)]^+^ complexes. Positions of the bands in IRMPD spectra reflect their IR spectra and can thus allow structural identification by comparison with theoretical spectra.^78^ It should be noted that due to the multiphotonic character of IRMPD spectra, the intensities of the individual bands can be distinctly different in comparison to single-photon theoretical spectra.^78^ The experimental spectra are presented for the 900 to 1800 cm^–1^ range and individually compared with theoretical IR spectra calculated by DFT calculations for possible isomers. By comparison, the isomer that was isolated and analyzed in the gas phase can then be matched with one or multiple of the theoretical possibilities allowing for direct identification.^79^–^84^

**Fig. 3:**
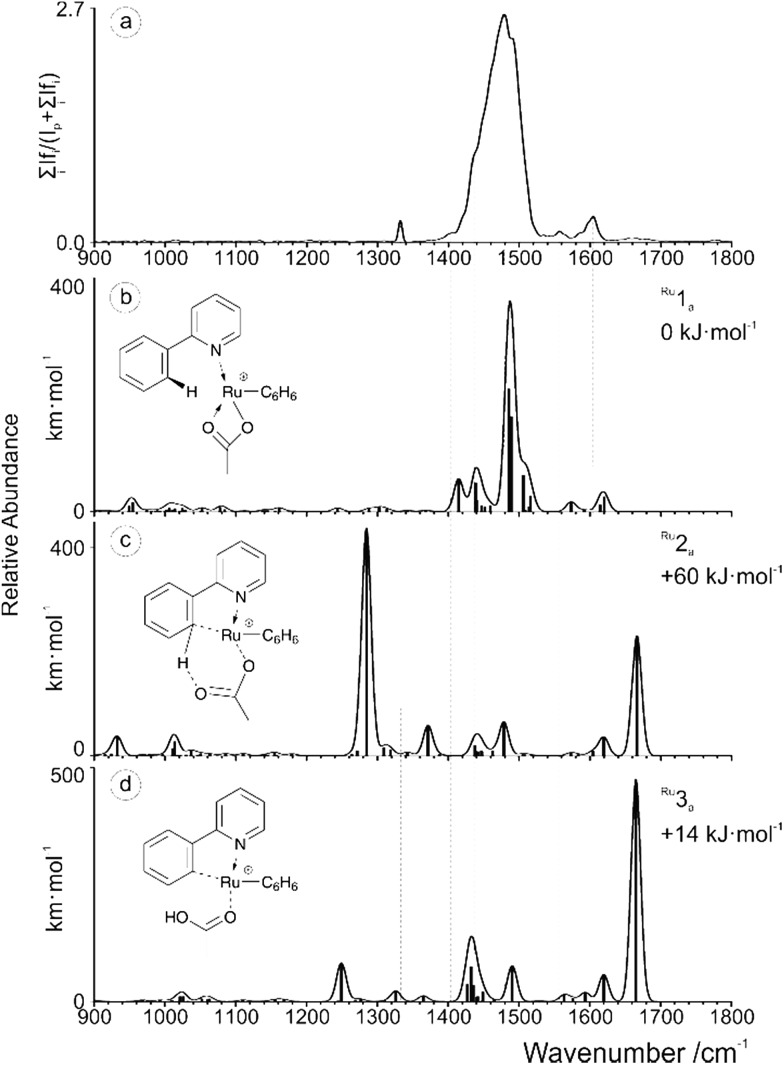
(a) IRMPD spectrum of the mass-selected [(2-PhPy)Ru(C_6_H_6_)(OAc)]^+^ complex and theoretical IR spectra of (b) ^Ru^1_a_, (c) ^Ru^2_a_ and (d) ^Ru^3_a_. The line spectra are presented along with a Gaussian function with fwhm = 16 cm^–1^. Corresponding calculated structures can be seen in Fig. S10 of the ESI.†

The experimental spectrum generated for the [(2-PhPy)Ru(C_6_H_6_)(OAc)]^+^ complex corresponds well to the theoretical spectrum of the initial ^Ru^1_a_ isomer (Fig. 3). In this structure the metal ion is coordinating to the acetate ligand through both oxygen atoms and the nitrogen of 2-PhPy while also interacting with the π-bonded electrons of benzene. This structure represents one of the initial reaction steps and was the most energetically stable to be found for this complex. Comparison of the spectra in Fig. 3a and b shows a strong principle match between the bands at 1480 cm^–1^ which can be theoretically assigned to symmetric and asymmetric C–O acetate stretching modes. Additional C–C double bond stretches of the 2-PhPy ligand (1557 and 1605 cm^–1^) correspond reasonably well to the experimental spectrum. The experimental bands at 1403 and 1435 cm^–1^ correspond to C–H bending vibrations within the acetate ligand. Fig. 3c shows the poor match between the IR spectrum of pre-activated complex ^Ru^2_a_ and experiment, especially for the key experimental C–O and C–C stretches.

Also, the characteristically shifted C–O and C=O vibrations (1284 and 1666 cm^–1^) are not seen experimentally. Similarly, the IR spectrum of the activated complex (Fig. 3d) contains only poorly matching bands in the key experimental range and the strong bands at 1250 cm^–1^ (combination of O–C–C antisymmetric stretch and OH bend) and 1665 cm^–1^ (metal coordinated C–O stretch) have no obvious match in the experimental spectrum.

An IRMPD spectrum for [(2-PhPy)_2_Cu(OAc)]^+^ is analogous to that of the ruthenium complex and also is in the excellent agreement with the theoretical spectrum of the non-activated intermediate ^Cu^1_a_ (Fig. S13 of the SI†). In particular the strong agreement between the dominant peaks centered around 1490 cm^–1^ gives a strong correlation with the theoretical bands corresponding to the symmetric and asymmetric C–O stretches of the bidentally coordinated acetate ligand. Monodentally coordinated acetate or acetic acid is characterized with strong bands at about 1200 (C–O vibration) and 1700 cm^–1^ (C=O vibration). These bands are not detected experimentally and therefore the corresponding isomers ^Cu^1_b_ and ^Cu^3_b_ are not generated.

**Fig. 4:**
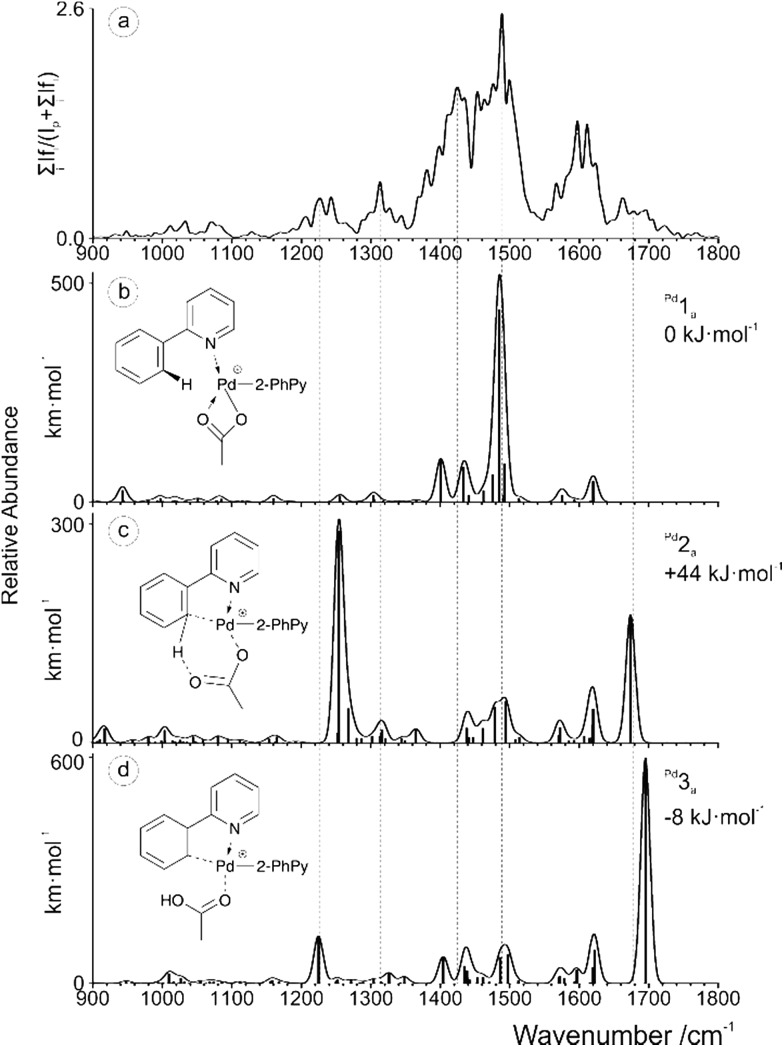
(a) IRMPD spectrum of the mass-selected [(2-PhPy)_2_Pd(OAc)]^+^ complex and theoretical IR spectra of (b) ^Pd^1_a_, (c) ^Pd^2_a_ and (d) ^Pd^3_a_. The line spectra are presented along with a Gaussian function with fwhm = 16 cm^–1^. Corresponding calculated structures can be seen in Fig. S12 of the ESI.†

The IRMPD spectrum obtained for [(2-PhPy)_2_Pd(OAc)]^+^ is shown along with corresponding theoretical spectra in Fig. 4 and it is considerably more complicated than the previous two examples. None of the calculated theoretical spectra provide an adequate match to the experimental results on their own. When this is the case, it is possible that either a non-considered isomer is being observed or contributions of two or more isomers are combining to produce a mixed result. Consideration of the calculated Pd potential energy surface (Fig. 2d) indicates that a mixed result is possible due to the low and comparable energies of ^Pd^1_a_ and ^Pd^3_a_.

Upon examining the relevant theoretical spectra clear similarities can be observed between the Pd complex and Cu/Ru equivalents. The spectrum appears to be dominated by the ^Pd^1_a_ non-activated isomer with large acetate C–O stretching bands at about 1485 cm^–1^. Well matching peaks at 1401 and 1433 cm^–1^ are from C–H bending of the acetic acid while the peaks at 1575 and 1621 cm^–1^ correspond to the C–C stretching modes of the 2-PhPy ligands. The experimental bands that have not been accounted for by ^Pd^1_a_, particularly those at 1230, 1310 and 1680 cm^–1^ could result from the presence of either the ^Pd^2_a_ or ^Pd^3_a_ isomer.

Given the energy profile observed in Fig. 2d, the presence of the ^Pd^3_a_ post-transfer isomer is highly probable. The theoretical bands for ^Pd^3_a_ that are observed at 1225 and 1695 cm^–1^ match particularly well with the experimental IRMPD spectrum. The 1225 cm^–1^ band results from a combination of a O–C–C asymmetric stretch and an O–H bend while the 1695 cm^–1^ peak corresponds to a C=O stretch involving the metal coordinated oxygen. This dual observation and interesting energy profile demonstrates the remarkable nature of the Pd example.

## Discussion

### Comparison of the experimental and theoretical results

We have repeated the calculations with different methods to better compare the theoretical and experimental energies, (Table 1).

**Table 1:** Calculated and experimental activation energies for AcOH loss

Method	Energy, kJ mol^–1^					
	Ru		Cu		Pd	
Experimental (CID)	113 ± 3		138 ± 5		65 ± 3	

	***E***_**diss**_	***E***_**TS**_	***E***_**diss**_	***E***_**TS**_	***E***_**diss**_	***E***_**TS**_
B3LYP-D2	142	64	156	139	71	83
B3LYP-D3	—	—	152	143	64	82
B3LYP	103	67	111	140	29	74

It can be seen that the dispersion corrections dramatically influence the AcOH elimination channel, whereas the activation barriers tend to only slightly change. According to Grimme's recent report, dispersion corrections performed on cations are likely an overestimate,^85^ which is reflected in our results.

For clarity in the discussion below we will be comparing the theoretical values obtained with the B3LYP-D2 method. For the ruthenium complex, a relatively small energy barrier for C–H activation was found. The subsequent acetic acid elimination requires almost 80 kJ mol^–1^ more energy.

The measured AE of the acetic acid loss thus reflects its dissociation limit from the ruthenium complex rather than the C–H activation barrier.

For the copper complex, the C–H activation energy barrier (*E*_rel_(^Cu^TS_1a–3b_) = 139 kJ mol^–1^) is on the order of the energy demand for the subsequent acetic acid loss (*E*_rel_(^Cu^4_a_ + AcOH) = 156 kJ mol^–1^). Elimination of AcOH is less abundant than elimination of 2-PhPy, although it is less energy demanding. It leads us to the conclusion that the C–H activation step is the limiting step and determines the appearance energy that we observe. The measured value of 138 ± 5 kJ mol^–1^ suggests that the theoretical values of the energy barrier as well as that of the dissociation limit are slightly overestimated (as in the ruthenium case).

Interpretation of the experimentally observed value for elimination of AcOH from the palladium complex is complicated by the fact that we study a mixture of activated and non-activated complexes. We believe that the experimentally measured value is largely dominated by the fragmentation of complexes with activated 2-phenylpyridine (elimination of AcOH at lower energies than it is predicted by theory (65 kJ mol^–1^*vs.* 79 kJ mol^–1^)). If the experimental value reflected the fragmentation of the complexes with non-activated 2-phenylpyridine the determined value would correspond to the energy barrier for C–H activation. The theoretical barrier is 83 kJ mol^–1^ which is 18 kJ mol^–1^ higher than the experimental value.

### Acid and substituent effects on the AEs of the carboxylic acid loss

Mass spectrometry is often used for studies of linear free energy correlations.^86^ Detailed investigation of organic reaction mechanisms, also in the gas phase, is often coupled with the study of Hammett plots associated with a given reaction. Therefore we decided to generate gas phase ions with the general formula [M(2-PhPy)(acid-H)(L)]^+^ and study their C–H activation, where “acid” stands for a series of *m*- and *p*- substituted benzoic acids and M = Ru, Cu and Pd. It was suggested that as few as five benzoic acids (*m*-NO_2_, *p*-NO_2_, *m*-Cl, H, *p*-CH_3_, *p*-CH_3_O) can provide a good reactive series to produce a Hammett plot.^87^ The inclusion of *p*-NO_2_ and *p*-CH_3_O derivatives enables a decision to be made about of what type of *σ* constants should be used (*σ*, *σ*^+^ or *σ*^–^).

To this end, we have studied a large series of LCu(OAc), 2-PhPy and acid mixtures in acetonitrile to generate the abovementioned intermediates and determined appearance energies for acid and 2-PhPy eliminations by CID (Table 2). We have selected copper complexes for detailed study because the activation energies observed experimentally can be associated with the C–H activation step.

**Table 2:** Measured activation energies in [Cu(acid-H)(2-PhPy)_2_]^+^ for two losses and the corresponding Hammett constants

Substituent	AE_acid_, kJ mol^–1^	AE_2-PhPy_, kJ mol^–1^	*σ*_*m*,*p*_
–H	149	157	0
*p*-NH_2_	148	153	–0.66
*p*-NO_2_	152	163	0.78
*p*-OCH_3_	155	157	–0.27
*p*-OH	148	156	–0.37
*p*-N(CH_3_)_2_	148	151	–0.83
*m*-OCH_3_	152	159	0.12
*m*-OH	148	157	0.12
*m*-NO_2_	157	162	0.71
*m*-Br	148	154	0.39

Correlation between AE's and the Hammett constants is rather poor, which arises from the very similar AE values, where the differences are on the order of the experimental error (3–5 kJ mol^–1^). We have therefore decided to investigate the relative cross sections of the fragmentation channels (*i.e.* losses of acids and 2-PhPy) that can be determined with a significantly greater precision than the AE values.^88^,^89^

**Fig. 5:**
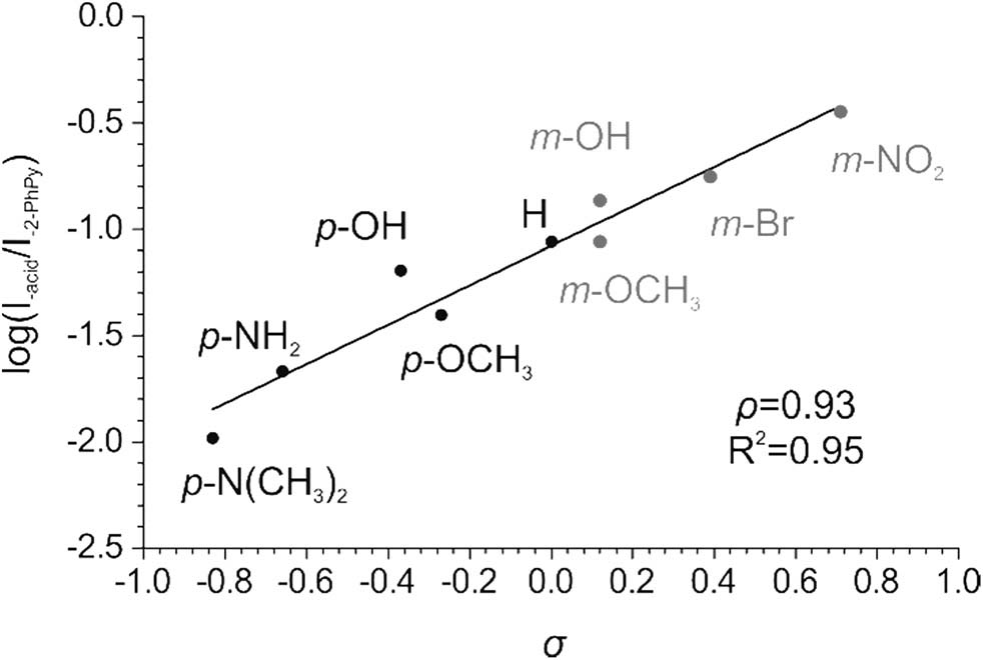
Hammett plot for the Cu-catalyzed C–H activation of 2-phenylpyridine showing the dependence of the logarithm of the branching ratio between acid and 2-phenylpyridine losses *vs.* the *σ* constant.

We have determined the relative abundances of the fragment ions [Cu(2-PhPy)((2-PhPy)-H)]^+^ and [Cu(2-PhPy)(acid-H)]^+^ at a collision energy where the branching ratio between the two fragmentation channels does not change with further collision energy increases (high energy plateau in the energy resolved CID plots, abundances were extracted from modelled CID curves, Fig. S16–S24†). We then plotted the logarithms of these fragmentation abundances against the corresponding Hammett *σ* parameter for each substituent. In the case of 2-PhPy loss, the substituent effect is negligible with *ρ* = –0.07 (Fig. S14 in ESI†). This means that electron withdrawing/donating substituents on a benzoate counter ion only marginally increase/decrease the binding energy between copper and 2-PhPy. Elimination of 2-PhPy has thus been used to anchor the substituent effect for the C–H activation step so that the logarithms of the branching ratios between the acid and 2-PhPy losses were used to construct the resulting Hammett plot (Fig. 5).

The dependence of the branching ratio logarithms on the acid substituent *σ* constants results in the *ρ* constant of 0.93. This value conveys (i) that electron-withdrawing substituents (stronger acids) accelerate the C–H activation step and (ii) a low ionic character of the transition state.

The first finding is in accordance with the experimental observation that addition of 4-nitrobenzoic acid to this type of reaction improves the C–H activation product yield.^90^ We note that the use of *σ*^+^ constants did not provide a better correlation (Fig. S15†). This suggests that the resonance effect from the substituent to the reaction center does not play a significant role.

The cause of the electronic effects is the structure of the concerted transition state in which hydrogen abstraction is assisted by the formation of the carbon–metal bond in the cyclic structure. The electron-withdrawing substituents at the carboxylate make the metal center more electrophilic and thus assist in the formation of the carbon–metal bond (see structures in Fig. 2). The theoretical cyclic transition structure with highly delocalized charge density is also in accordance with the small value of *ρ*.

**Fig. 6:**
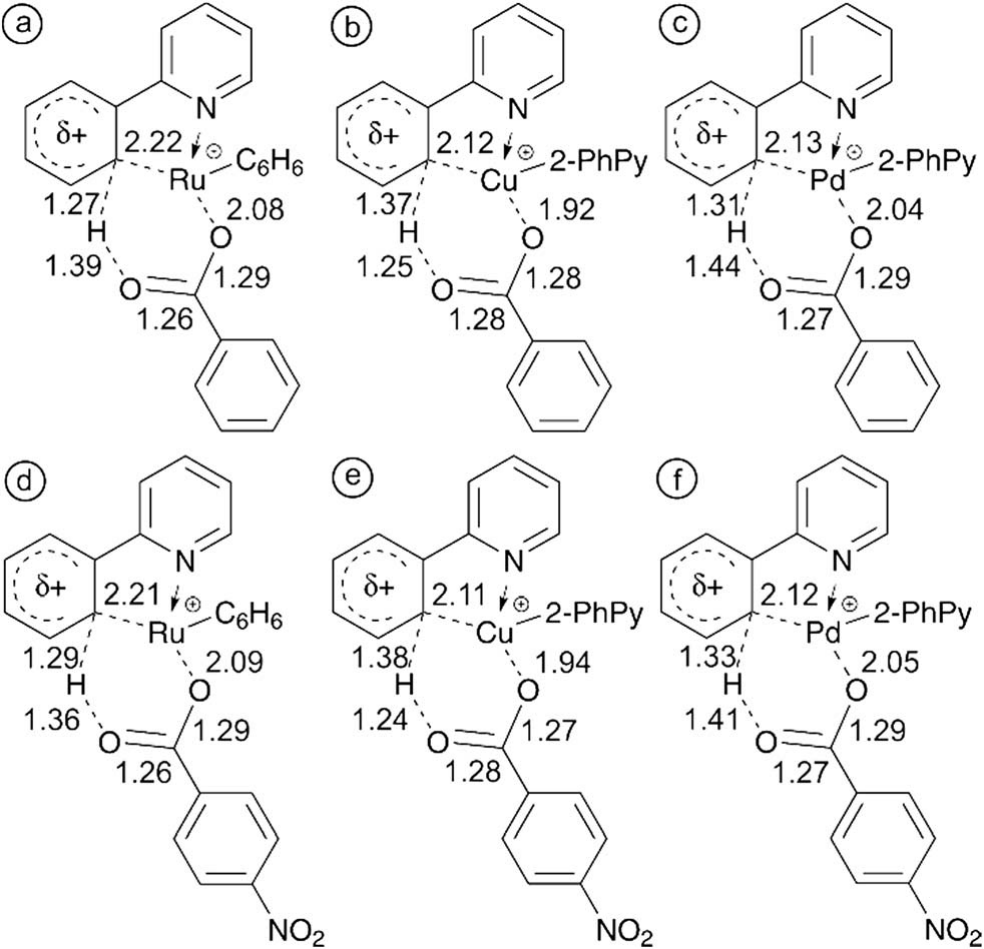
6-membered transition structures showing key bond lengths for (a) ^Ru^TS_2a–3a_, (b) ^Cu^TS_1a–3b_ and (c) ^Pd^TS_2a–3a_ with a benzoate counter ion and (d) ^Ru^TS_2a–3a_, (e) ^Cu^TS_1a–3b_, and (f) ^Pd^TS_2a–3a_ with a 4-nitrobenzoate counter ion. All distances are in Å.

To validate the key role of the formation of a metal–carbon bond in the cyclic transition structure, we have reoptimized the structures for C–H activation using benzoate and 4-nitrobenzoate as the counter ion. Fig. 6 shows the bond lengths in each of the six-membered transition structures allowing us to compare the electronic effect from the electron withdrawing NO_2_ group on the mechanism of C–H activation.

With all three of the metals, the metal–carbon distance decreases while the metal–oxygen and carbon–hydrogen distances both increase. These observations are in line with the theorized increasingly electrophilic metal center. Complete potential energy surfaces for each of these processes can be found in the ESI (Fig. S25–S30†). In each case, the presence of NO_2_ reduced the energy required for 4-nitrobenzoic acid fragmentation in comparison with benzoic acid. This further supports the experimental observation of an increasingly electrophilic metal center. For additional clarity the study was also performed with the strongly electron withdrawing trifluoroacetate counter ion. Comparisons of the TS bond lengths for acetate and trifluoroacetate and complete potential energy surfaces can be found in the ESI (Fig. S31–S34†).

## Conclusions

We have shown that carboxylate assisted C–H activation of 2-phenylpyridine can be studied in the gas phase with ruthenium, copper, and palladium catalysts.

Using infrared multiphoton dissociation spectroscopy, we have demonstrated that we can isolate ruthenium acetate and copper acetate complexes with non-activated 2-phenylpyridine and induce the C–H activation upon the collisional activation. In the case of palladium acetate complexes, we have observed a mixture of complexes with both non-activated and C–H activated 2-phenylpyridine ligands. We have determined experimental energies required for the C–H activation and subsequent acetic acid elimination. Further analysis revealed that the determined energy for the ruthenium and palladium complexes chiefly reflects the binding energy of acetic acid to the metal center after the C–H activation step, whereas the energies determined for copper complexes reflect the activation energies for the C–H activation steps.

DFT calculations were employed to determine potential energy surfaces for C–H activation and acetic acid loss with each metal. The complexes and transition structures along the reaction coordinate were very similar but with vastly differing relative energies. The Ru and Pd examples included a structure where the transferring proton was partly interacting with AcO^–^ primed for easy transfer. We located six-membered transition states involving the 2-PhPy, AcO^–^ and metal as was previously hypothesized.

The C–H activation step was also studied using Hammett plots for C–H activation in copper complexes with a series of substituted benzoates. The results clearly showed that electron-withdrawing substituent at the benzoates (*i.e.* use of stronger acids) accelerate the C–H activation step. The explanation can be found in the cyclic nature of the transition structure, where the C–H bond dissociation is assisted by the formation of the carbon–metal bond. Carboxylates derived from stronger acids support more the formation of the carbon–metal bond.

## Materials and methods

### Chemicals

All of the chemicals used in the experiments below were purchased from Sigma Aldrich and used without further purification.

### Mass-spectrometric and IRMPD experiments

The experiments were performed on a Finnigan LCQ ion trap mass spectrometer equipped with an ESI source. The studied solutions were prepared using acetonitrile as a solvent and were introduced to the ESI source through a fused-silica capillary by a syringe pump at a rate of 3 μL min^–1^. Nitrogen was used as a nebulizing and drying gas throughout the experiments. The operating conditions were set as follows (if not otherwise mentioned): spray voltage 4.5 kV, capillary voltage 0 V, tube lens offset 0 V, heated capillary temperature 150 °C. All of the mass spectra were recorded from *m*/*z* 50 to *m*/*z* 1000. Collision-induced dissociation (CID) of mass selected precursor ions was achieved by RF-excitation of the ions within the ion trap where He was used as the buffer gas and collision partner. The collision energy was optimized for each experiment and using Schröder's calibration method,^91^ the appearance energies for the ions of interest were derived.

Infrared multiphoton dissociation (IRMPD) spectra were recorded on a Bruker Esquire 3000 ion trap mass spectrometer coupled to the free electron laser at CLIO (Centre Laser Infrarouge Orsay, France).^92^ The solutions used for IRMPD measurements were prepared in the same way as those used in the ESI-MS experiments described above. The ions of interest were generated with an electrospray source, mass selected and stored in the ion trap. The free electron laser (FEL) operating at 44 MeV electron energy provided light in the 900–1800 cm^–1^ range and with a spectral resolution (full width at half-maximum) in the range of 15–20 cm^–1^.^93^,^94^ Fragmentation of the ions was induced by 5–10 laser macropulses of 8 μs directed into the ion trap. The IRMPD spectra are given by dependence of the fragmentation intensities on the wavelength of the IR light. At a given wavelength each point in a raw spectrum is obtained by the evaluation of 4 mass spectra and each mass spectrum is an average of 5 measurements. The IRMPD spectra are not corrected for the power of the free-electron laser, which was dropping towards high wavenumbers (see the ESI†).

### DFT calculations

Density functional theory calculations were performed with the Gaussian 09 suite. The B3LYP method was used for all calculations. Two different combinations of basis sets were applied. With acetate and trifluoroacetate counter ions, the cc-pVTZ basis set was used for O, N, C and H and where appropriate the cc-pVTZ-pp basis set was applied for Ru, Cu and Pd. With benzoate and 4-nitrobenzoate counter ions, the 6-31G* basis set was used for O, N, C and H and the SDD basis set was applied for Ru, Cu and Pd. All of the structures have been fully optimized and established as genuine minima or transition states on the appropriate potential energy surfaces as confirmed by analysis of the corresponding Hessian matrices. Frequency analysis also enabled calculation of thermochemical correction and energies are subsequently reported as zero point energies. As the relative position of ligands is critical to the progression of the reaction, corrections for dispersion were included. Except where clearly stated the D2 version of Grimme's dispersion,^95^ that has previously been defined for the B3LYP method, was enabled for all calculations. The D3 version of Grimme's dispersion was also employed to a limited degree for comparison.^96^ All optimized structures and energies can be found in the ESI.† Corrections for the basis set superposition error (BSSE) were included for dissociations. Transition state structures were confirmed by IRC calculations. Pd and Ru were calculated as singlet state complexes while Cu was a doublet. All possible spin states were calculated with the B3LYP method and D2 empirical dispersion with the 6-31+G(d,p) basis set on O, N, C and H and LanL2DZ on the metal. The most stable ones were further optimised at the final level of theory.

The theoretical IR spectra were corrected with a scaling factor of 0.985.

## Supplementary Material

Supplementary information

Supplementary information
