# Supplementary material for: Carboxylate-assisted C–H activation of phenylpyridines with copper, palladium and ruthenium: a mass spectrometry and DFT study
Source: Chem Sci. 2015 Jul 1;6(10):5544–53. doi: 10.1039/c5sc01729g (PMC5949854; doi:10.1039/c5sc01729g)
Supplement: Supplementary file 2 [file SC-006-C5SC01729G-s002.pdf]

Electronic Supplementary Material (ESI) for Chemical Science.
This journal is © The Royal Society of Chemistry 2015

Supporting Information for: "Carboxylate-Assisted C-H Activation of Phenylpyridines: A Mechanistic Study"

by Andrew Gray, Alexandra Tsybizova and Jana Roithova*

Department of Organic Chemistry, Faculty of Science, Charles University in Prague, Hlavova 2030/8, 12843 Prague 2 (Czech Republic)

Table of contents

1. **ESI-MS studies** 1
2. **Calculated structures** 5
3. **IRMPD studies** 6
4. **Hammett studies** 7
5. **Benzoic Acid and 4-NitroBenzoic Acid DFT Study** 12
6. **Trifluoroacetic acid DFT study** 18
7. **Laser power dependence** 23

1. ESI-MS studies

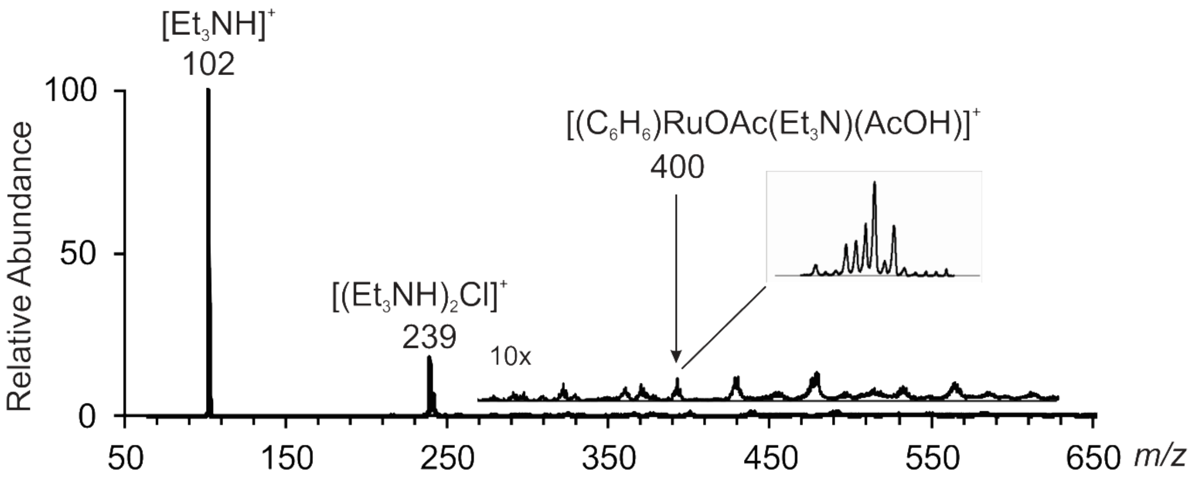

Figure S1. a) ESI-MS spectrum of the mixture of Ru-dimer complex, acetic acid and triethylamine in acetonitrile

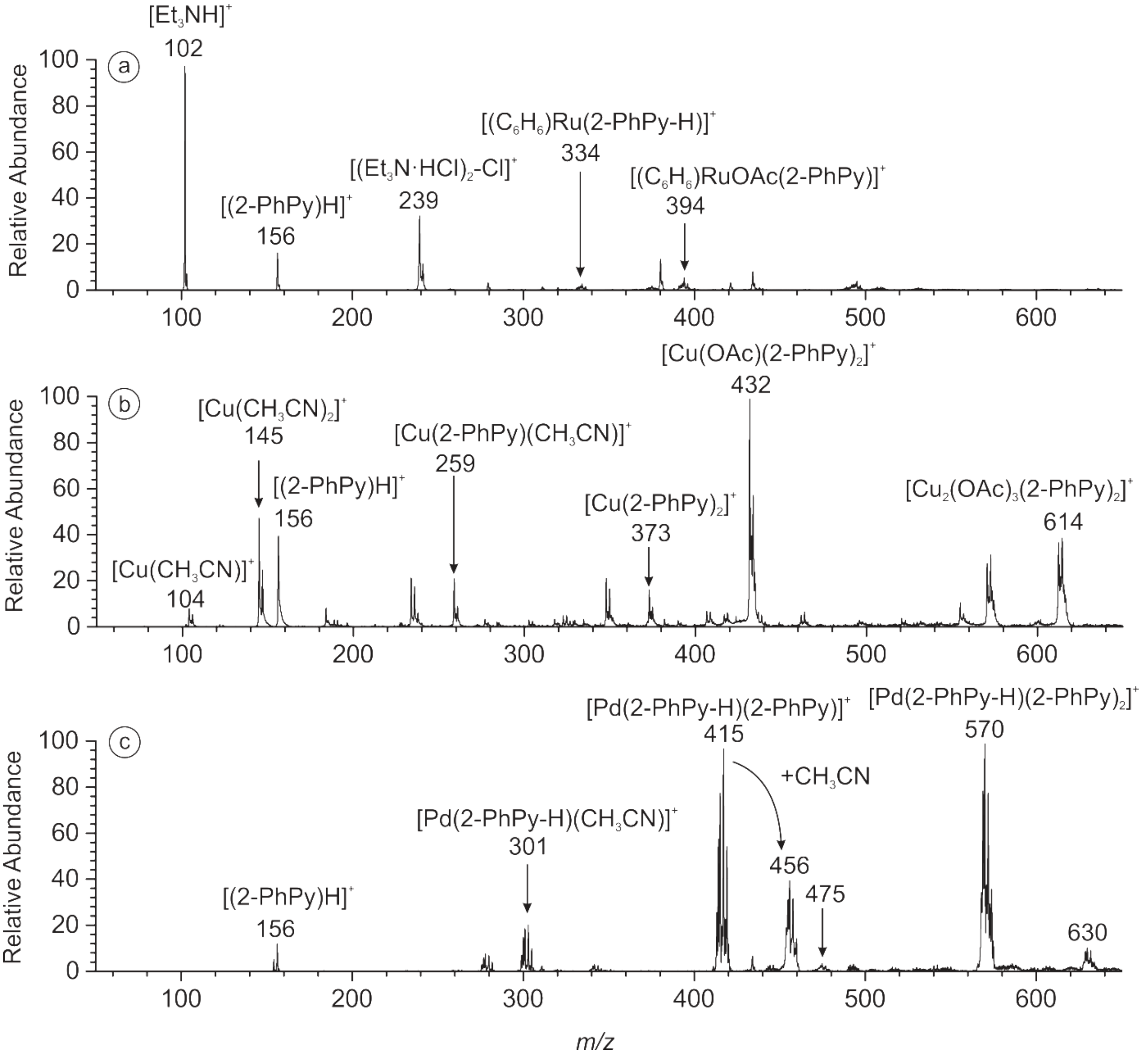

Figure S2. a) ESI-MS spectrum of Ru-dimer complex, acetic acid, triethylamine and 2-phenylpyridine in acetonitrile; b) ESI-MS spectrum of $Cu(OAc)_2$ and 2-PhPy in acetonitrile; c) ESI-MS spectrum of $Pd(OAc)_2$ and 2-PhPy in acetonitrile.

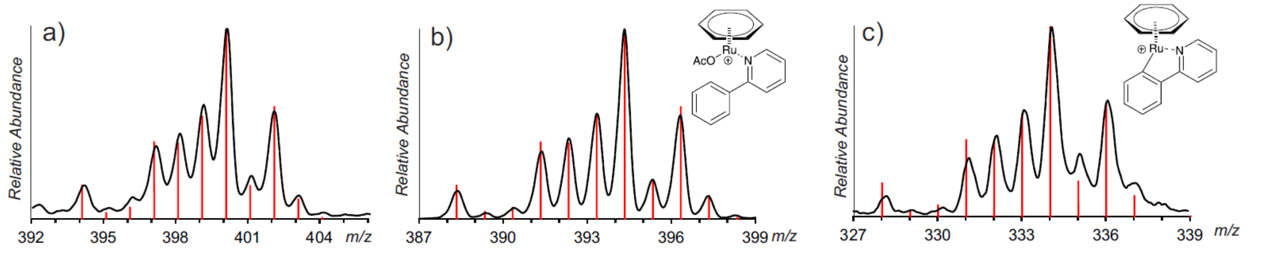

Figure S3. Isotope patterns of the investigated Ru-ions presented in the main paper (the red bars show the theoretical isotope patterns corresponding to the suggested ionic structures). (a) $[(C_6H_6)RuOAc(Et_3N)(AcOH)]^+$ (b) $[(C_6H_6)RuOAc(2\text{-}PhPy)]^+$ (c) $[(C_6H_6)Ru((2\text{-}PhPy)\text{-}H)]^+$.

In order to better understand the behavior of the reaction mixture for Ru(II) catalysis we have studied the dependence of the 2-phenylpyridine concentration on the formation of ions in the gas phase. The results are shown in Figure S4. For the sake of simplicity we kept the concentration of the catalytic solution the same in all of the solutions tested (10^{-3} M, where the ruthenium dimer, acid and the base were mixed in 1:1:1 ratio), and only varied the amount of 2-PhPy.

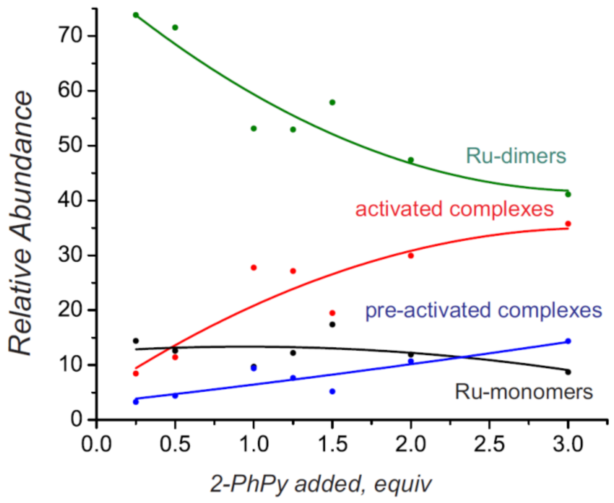

Figure S4. Dependence of the relative abundance of various Ru-clusters on the amount of 2-PhPy in the solution.

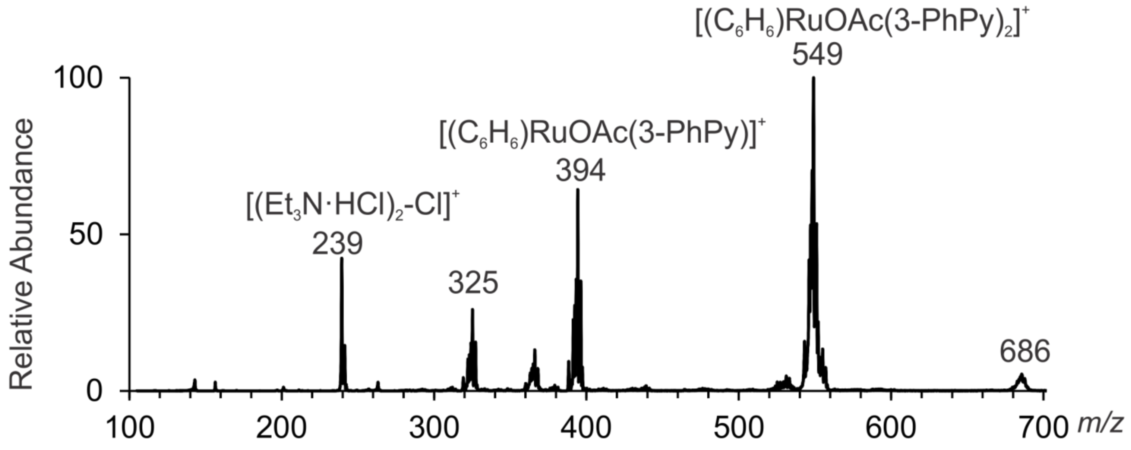

Figure S5. ESI-MS spectrum of Ru-dimer complex, acetic acid, thriethylamine and 3-phenylpyridine in acetonitrile.

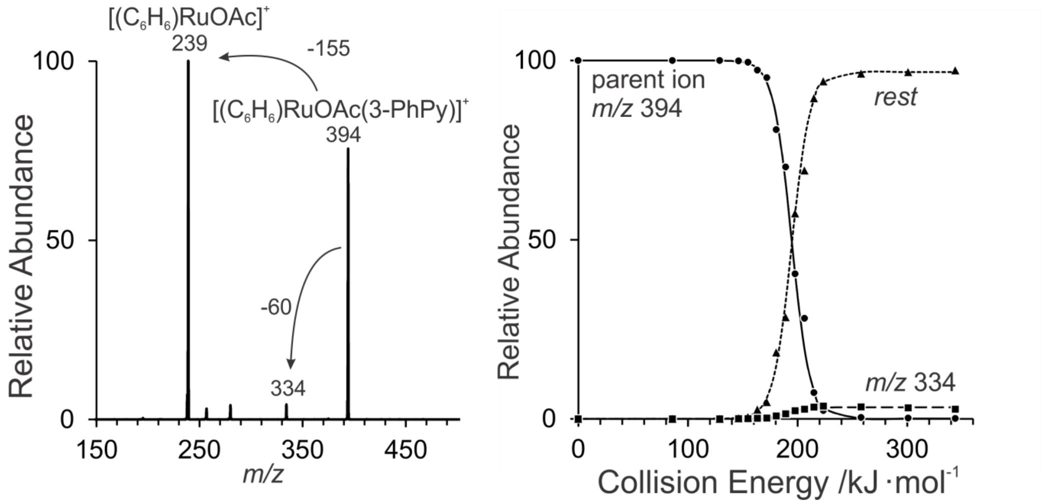

Figure S6. CID spectrum of the mass selected peak at *m/z* 394 and its breakdown curve. *AE* for AcOH loss = 173 ± 3 kJ•mol^{-1}. *AE* for 3-PhPy loss = 178 ± 3 kJ•mol^{-1}

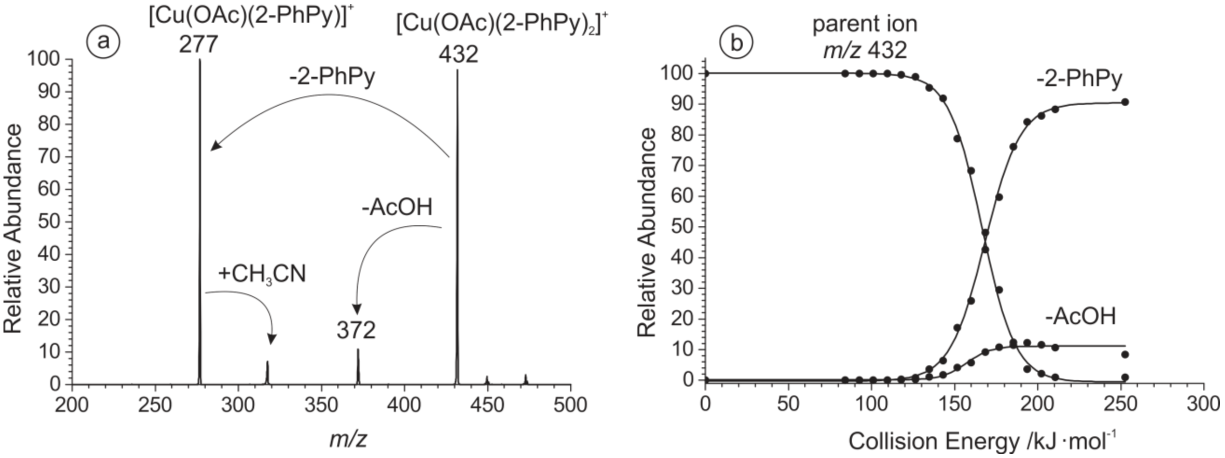

Figure S7. The CID spectrum and the breakdown curve for the mass selected peak at m/z 432. AE for the AcOH loss: 138 ± 5 kJ·mol^{-1}, AE for the 2-PhPy loss: 147 ± 5 kJ·mol^{-1}.

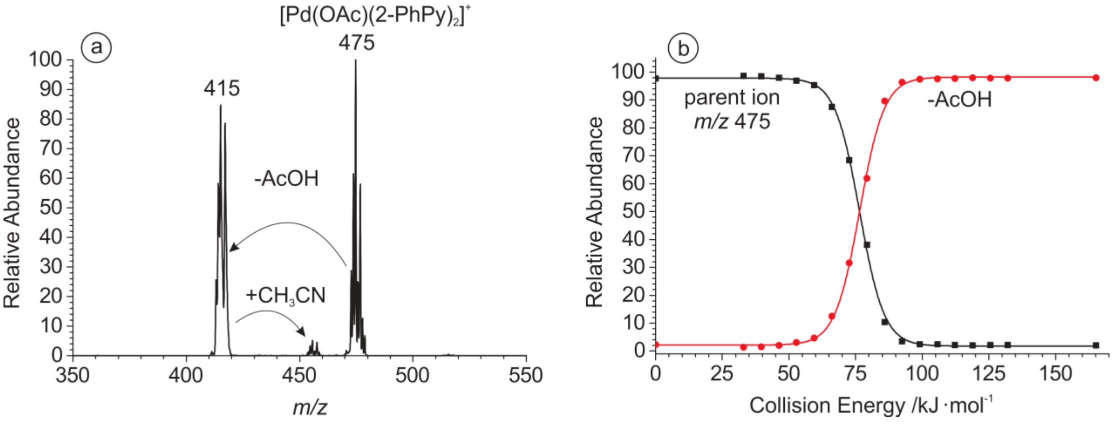

Figure S8. The CID spectrum of the mass selected peak at *m/z* 475 and its breakdown curve. *AE* for AcOH loss: 65 ± 3 kJ·mol^{-1}.

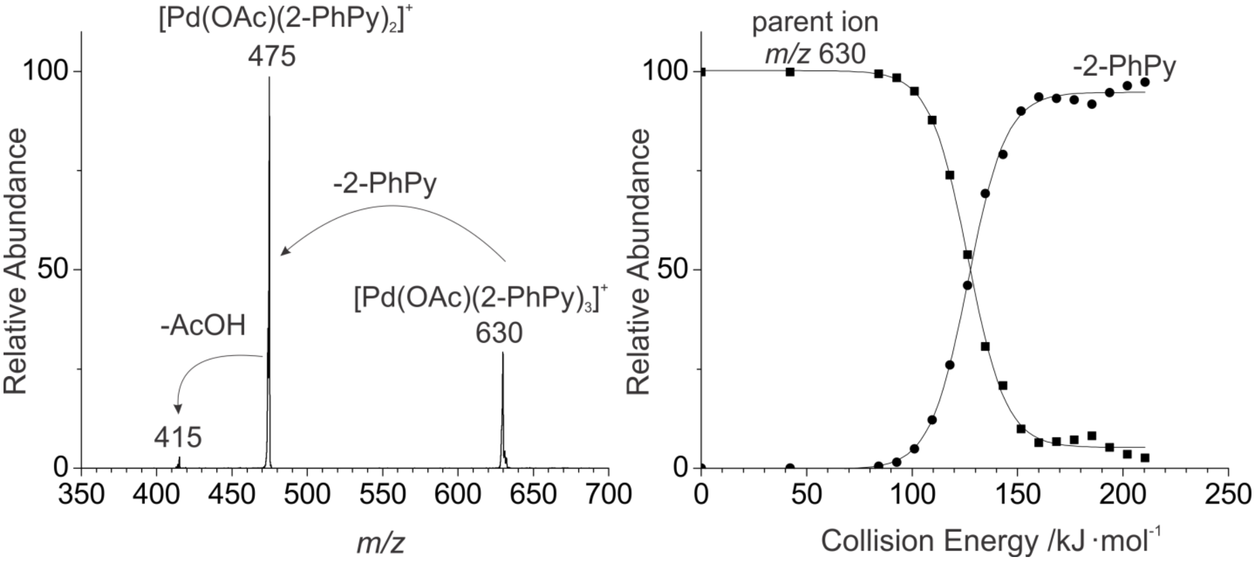

Figure S9. CID spectrum for the mass selected peak at *m/z* 630 taken at collision energy = 135 kJ·mol^{-1} and its breakdown curve. Appearance energy for 2-PhPy loss is 108 ± 3 kJ·mol^{-1}.

2. Calculated structures

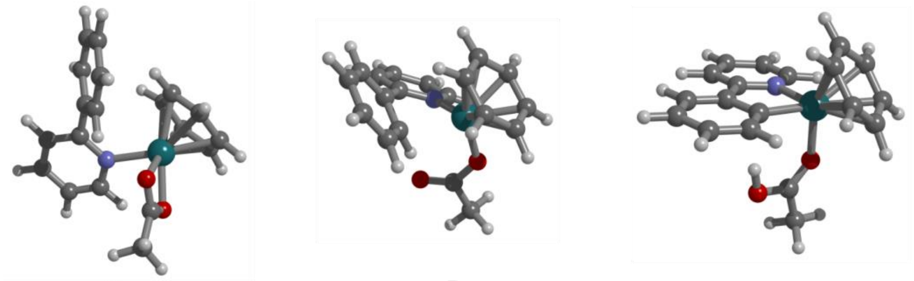

$^{Ru}1_a$ $^{Ru}2_a$ $^{Ru}3_a$

Figure S10. Selected optimized structures for ruthenium catalysis.

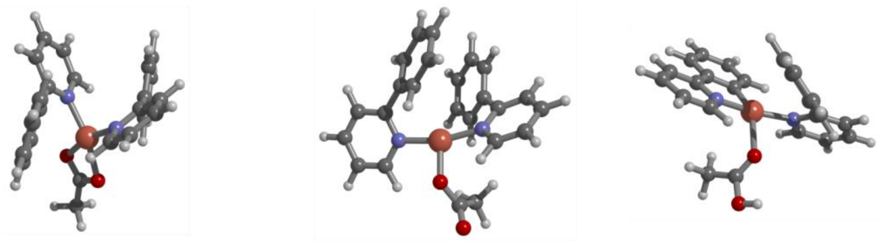

$^{Cu}1_a$ $^{Cu}1_b$ $^{Cu}3_b$

Figure S11. Selected optimized structures for copper catalysis.

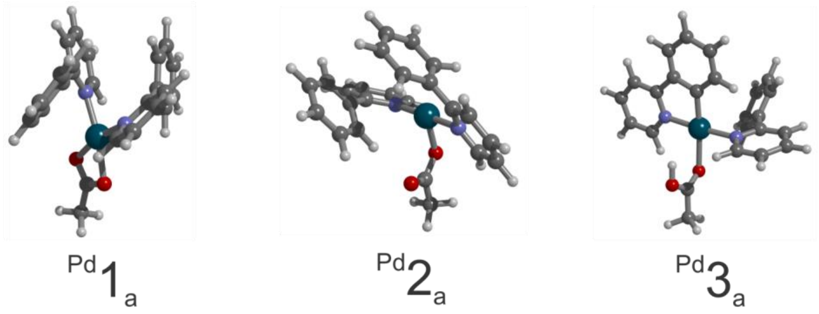

Figure S12. Selected optimized structures for palladium catalysis.

3. IRMPD studies

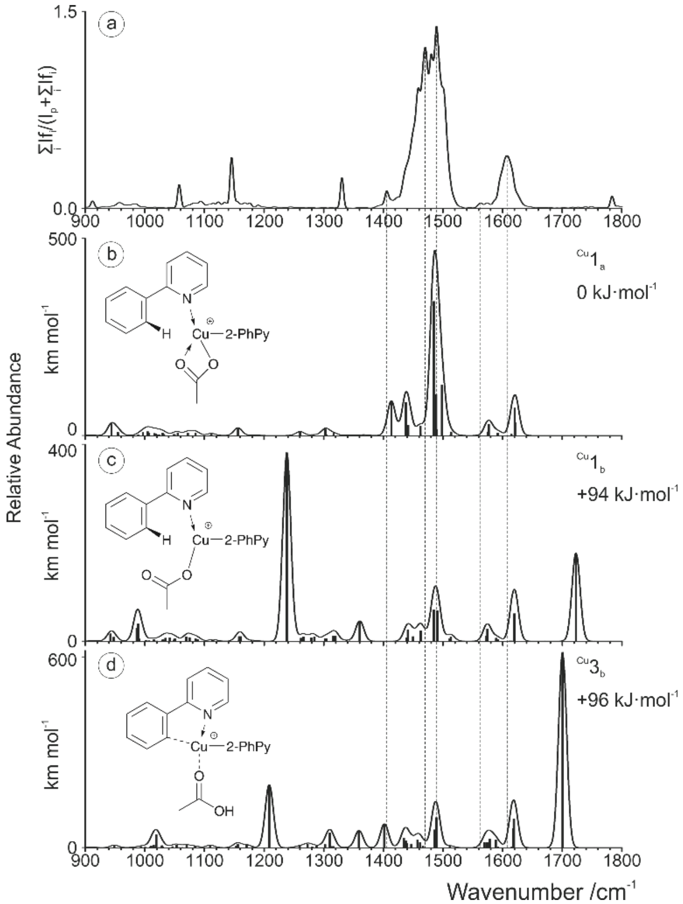

Figure S13. a) IRMPD spectrum of the mass-selected $[(2\text{-PhPy})_2Cu(OAc)]^+$ complex and theoretical IR spectra of b) $^{Cu}1_a$, c) $^{Cu}1_b$ and d) $^{Cu}3_b$. The line spectra are presented along with a Gaussian function with fwhm = 16 cm^{-1}. Corresponding calculated structures can be seen in Figure S11.

4. Hammett studies

We decided to use plateau intensities, and the branching ratio between acid and 2-PhPy losses as a function of the *σ* constant, for the final interpretation of the results as the latter approach showed the highest accuracy interpretation of the results.

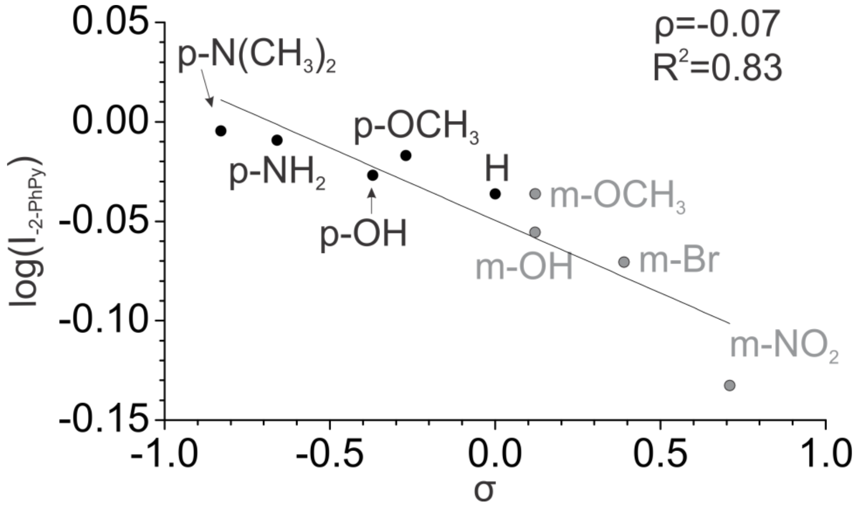

Figure S14. Hammett plot for Cu-catalyzed C-H activation of 2-phenylpyridine showing the dependence of the logarithm of the relative intensity of 2-PhPy loss at plateau vs *σ* constant.

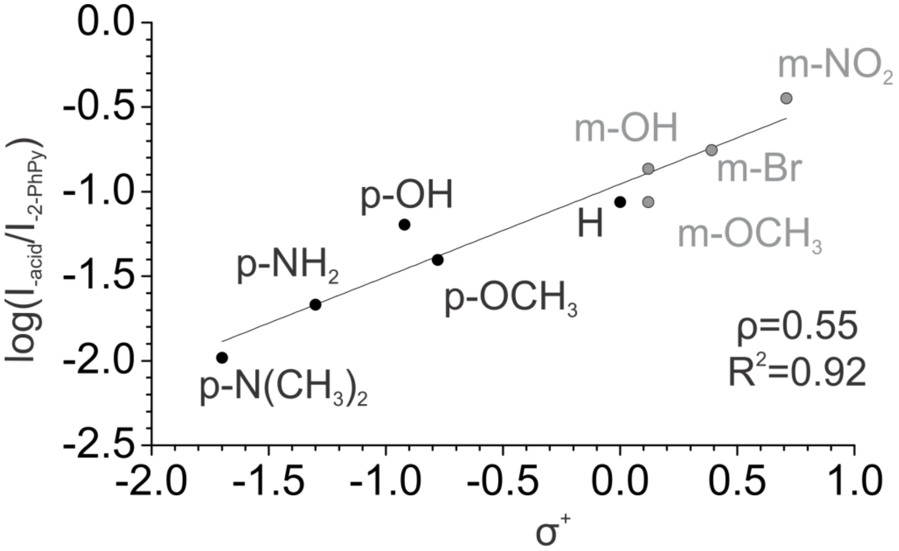

Figure S15. Hammett plot for Cu-catalyzed C-H activation of 2-phenylpyridine showing the dependence of the logarithm of the branching ratio between acid and 2-phenylpyridine losses vs the *σ*+ constant.

The branching ratio was determined from the fitted CID curves (Figures S16-S24).

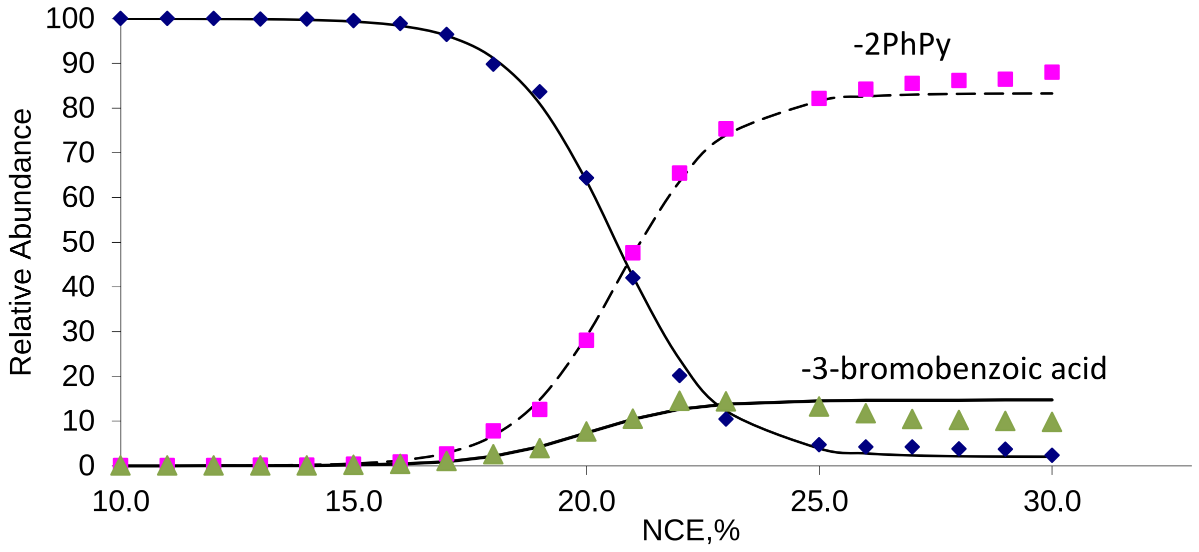

Figure S16. The breakdown curve for the mass selected peak at *m/z* 572 (3-bromobenzoic acid derivative).

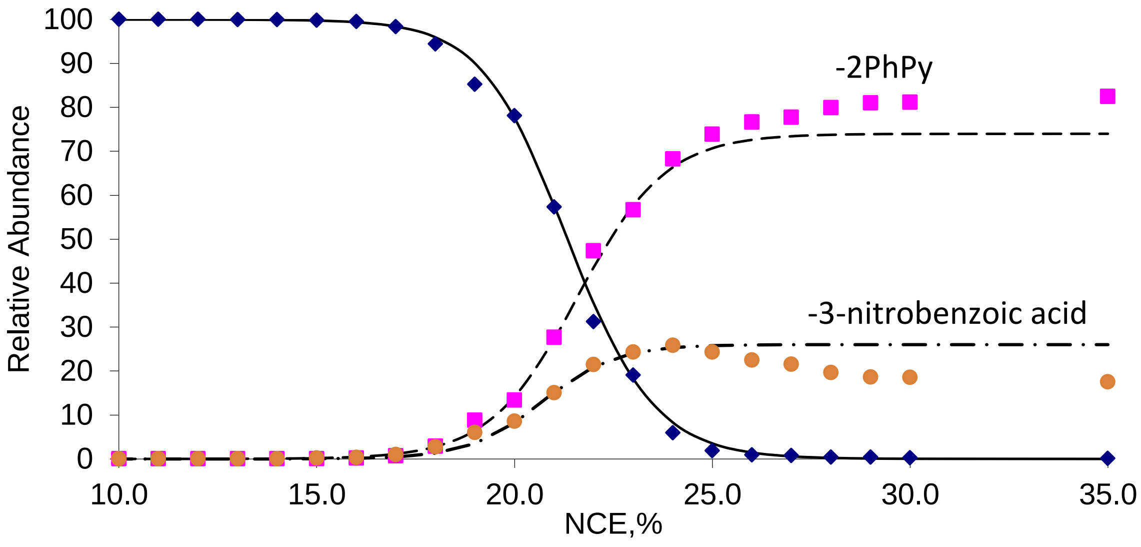

Figure S17. The breakdown curve for the mass selected peak at *m/z* 539 (3-nitrobenzoic acid derivative).

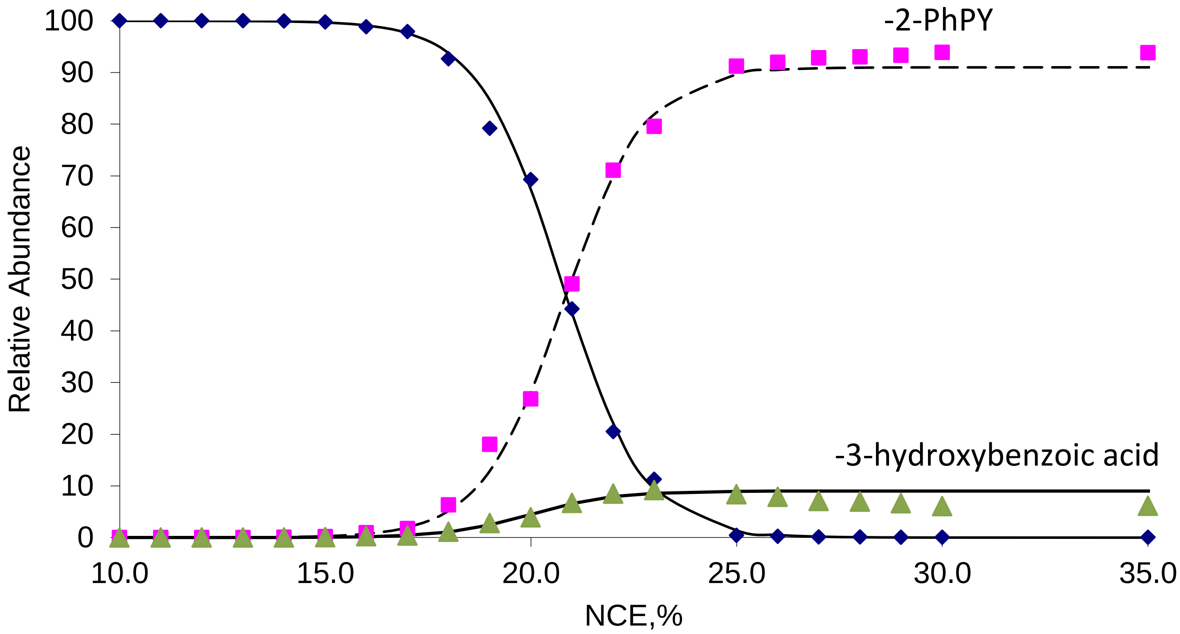

Figure S18. The breakdown curve for the mass selected peak at *m/z* 510 (3-hydroxybenzoic acid derivative).

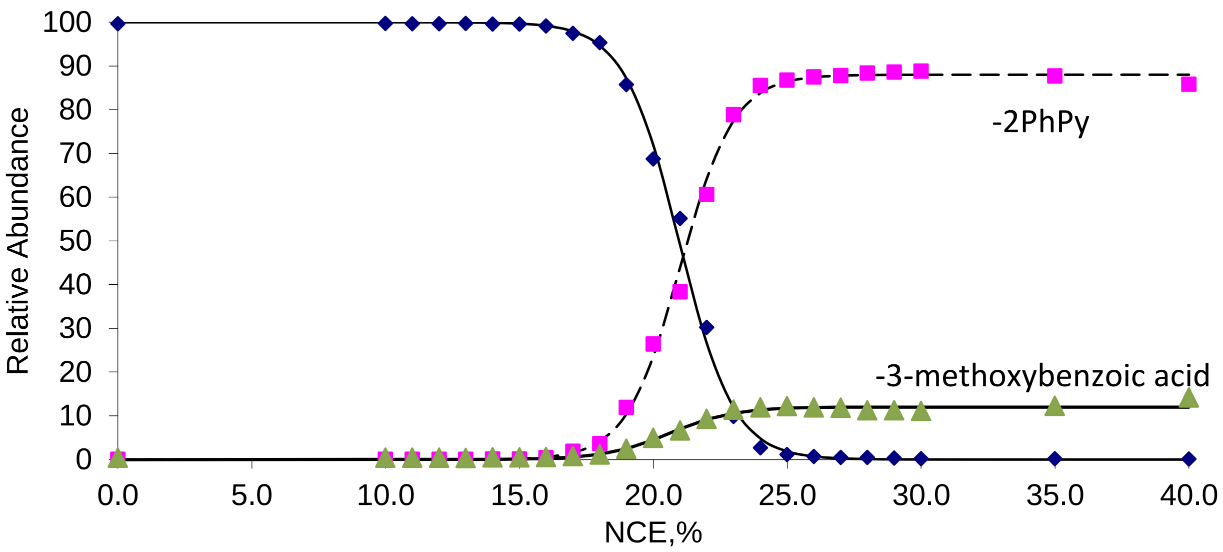

Figure S19. The breakdown curve for the mass selected peak at *m/z* 524 (3-methoxybenzoic acid derivative).

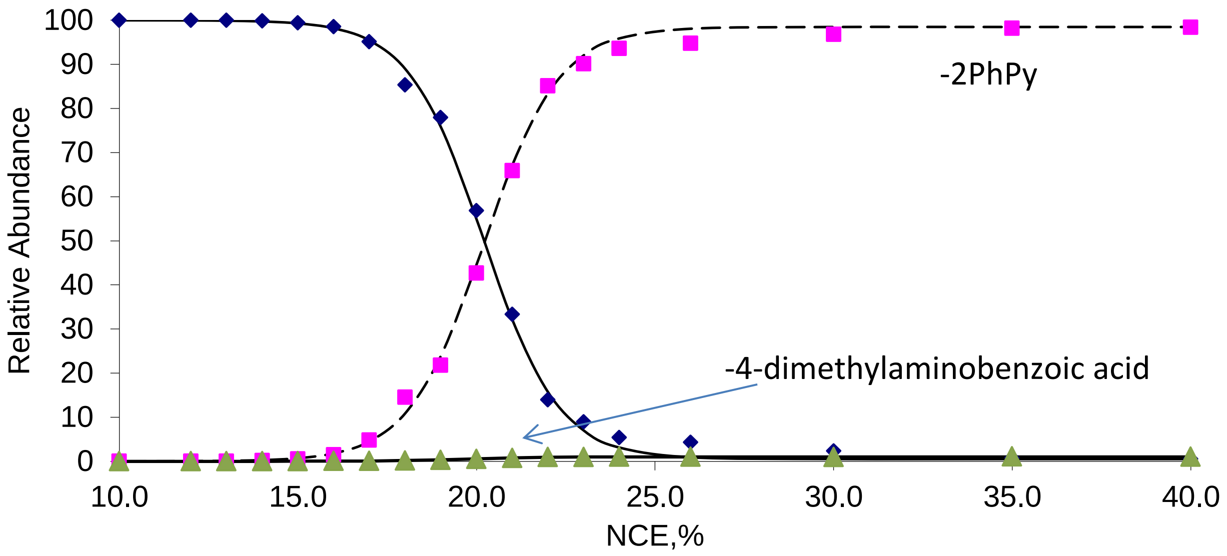

Figure S20 The breakdown curve for the mass selected peak at *m/z* 537 (4-dimethylaminobenzoic acid derivative).

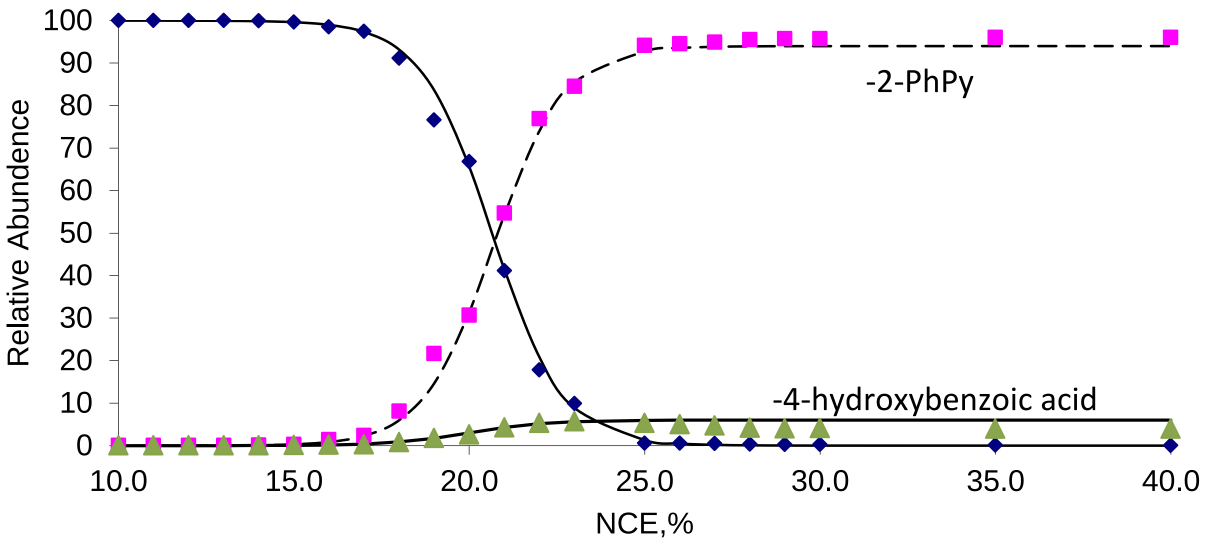

Figure S21. The breakdown curve for the mass selected peak at *m/z* 510 (4-hydroxybenzoic acid derivative).

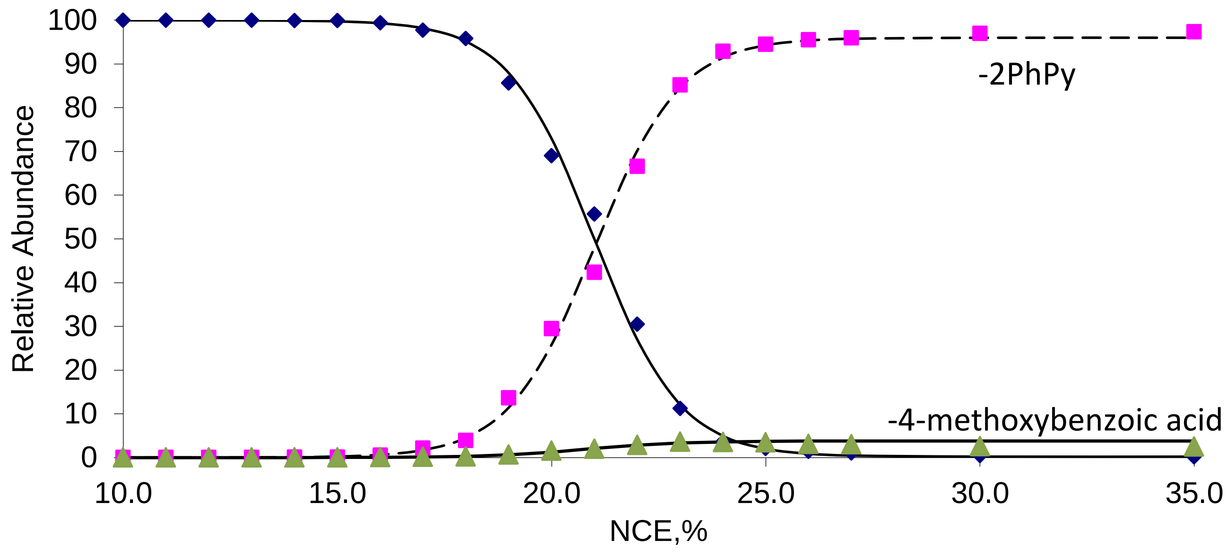

Figure S22. The breakdown curve for the mass selected peak at *m/z* 524 (4-methoxybenzoic acid derivative).

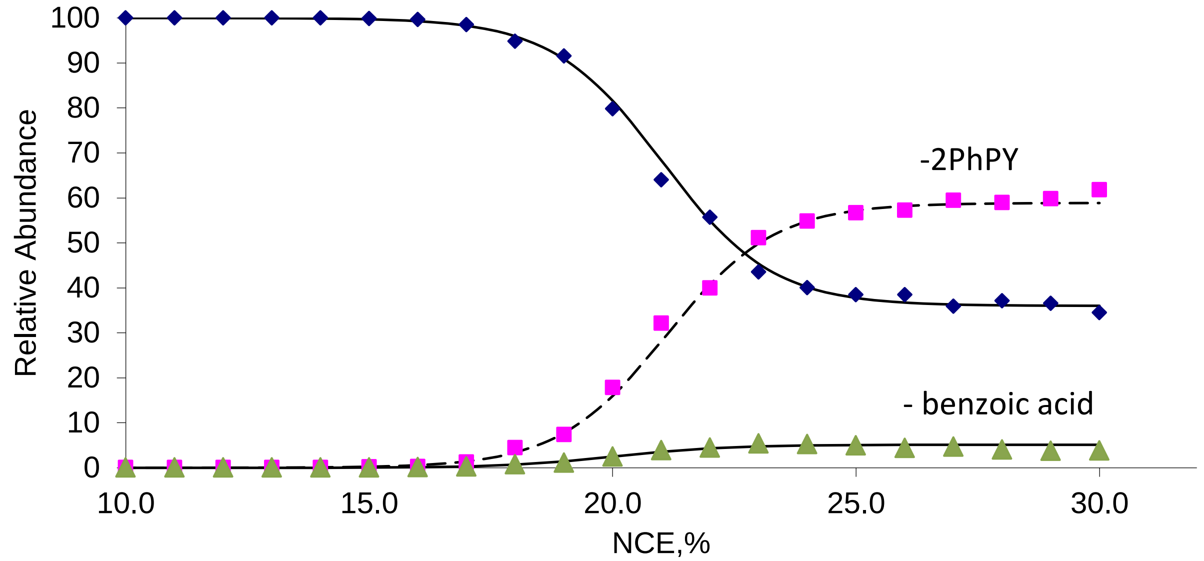

Figure S23. The breakdown curve for the mass selected peak at *m/z* 494 (benzoic acid derivative).

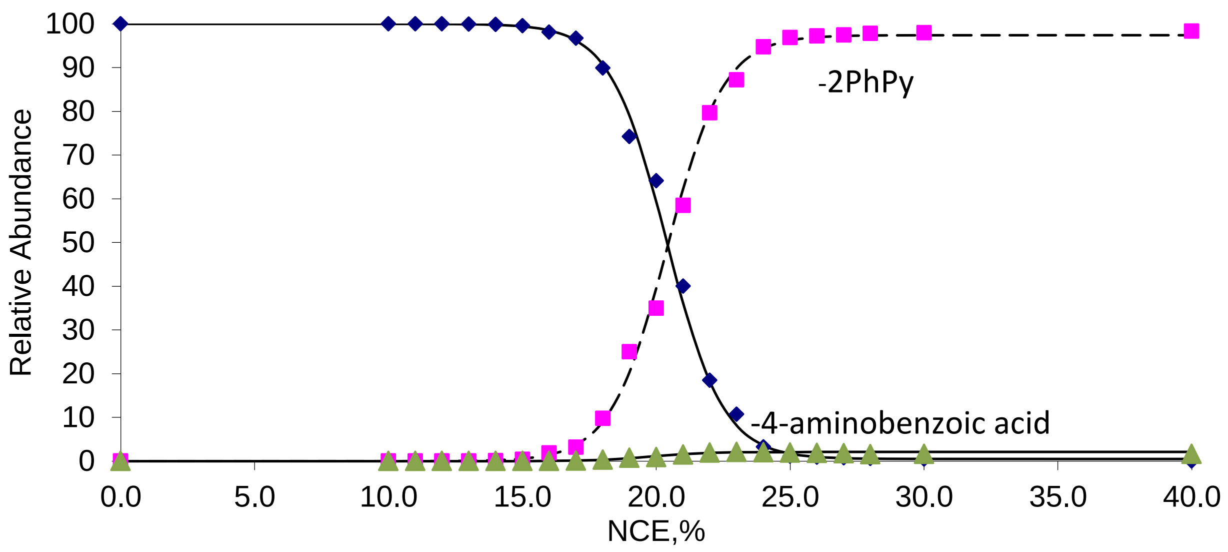

Figure S24. The breakdown curve for the mass selected peak at *m/z* 509 (4-aminobenzoic acid derivative).

5. Benzoic Acid and 4-NitroBenzoic Acid DFT Study

The calculated potential energy surfaces with benzoate and 4-nitrobenzoate counter ions in place of acetate for all three of the catalytic processes previously shown. Equivalent structures were located and each of the reactions appears likely to proceed in the same manner as previously observed with the acetate counter ion. Structures are thus labelled in the same format. Calculations were performed with the B3LYP method and D2 empirical dispersion along with the 6-31G* (O, N, C, H) and SDD (Ru, Cu, Pd) basis sets.

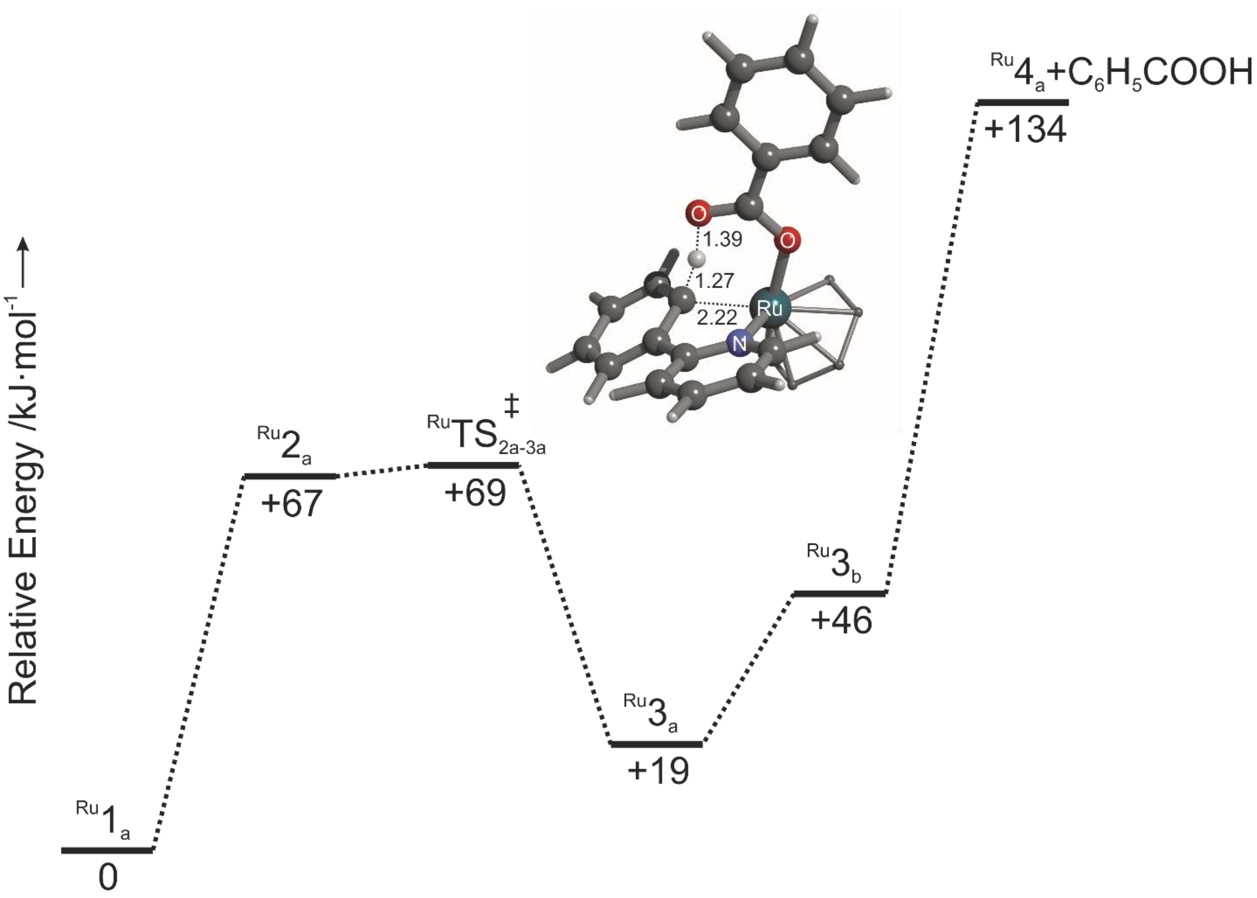

Figure S25. Zero point potential energy surface (B3LYP/6-31G*:SDD(Ru)) for the Ru assisted C-H activation of 2-PhPy with a $C_6H_5COO^-$ counter ion. Representations of structures are shown in Figure 2. All distances are in Å.

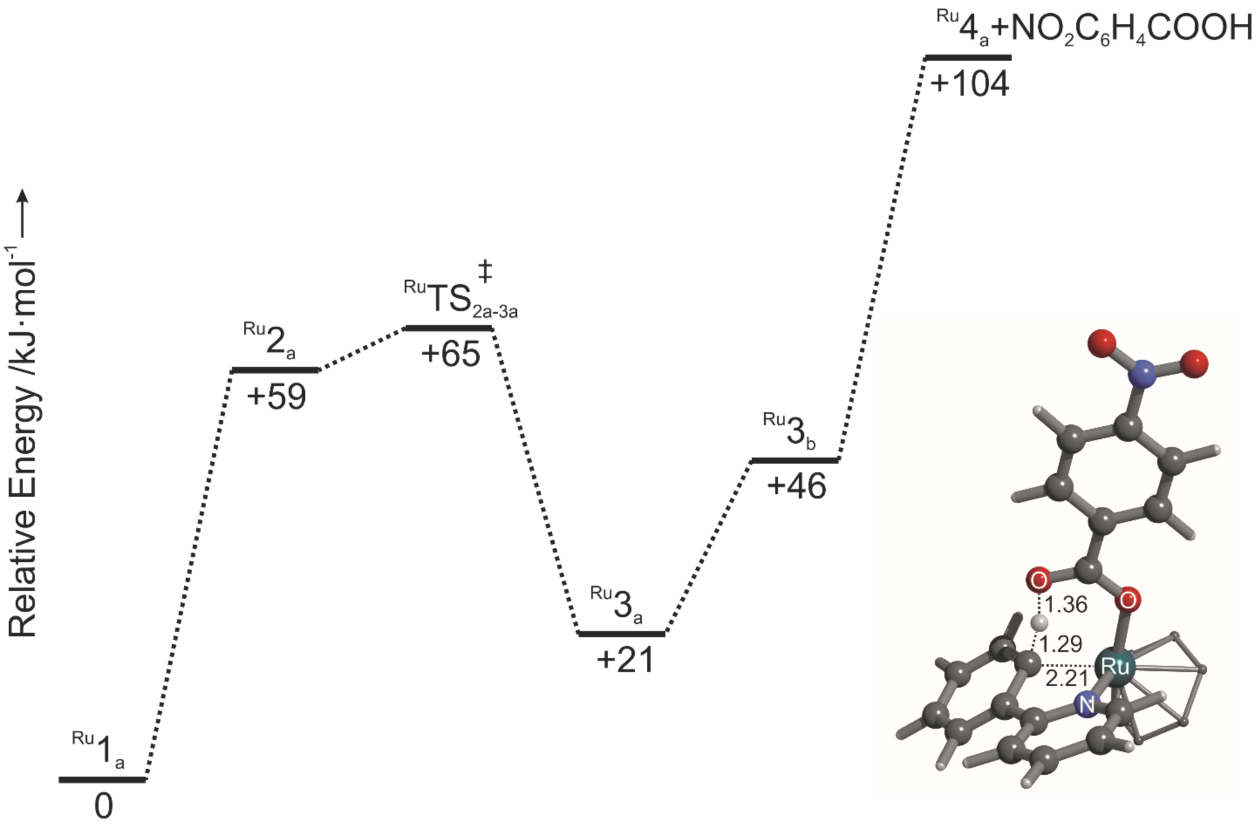

Figure S26. Zero point potential energy surface (B3LYP/6-31G*:SDD(Ru)) for the Ru assisted C-H activation of 2-PhPy with a $NO_2C_6H_4COO^-$ counter ion. Representations of structures are shown in Figure 2. All distances are in Å.

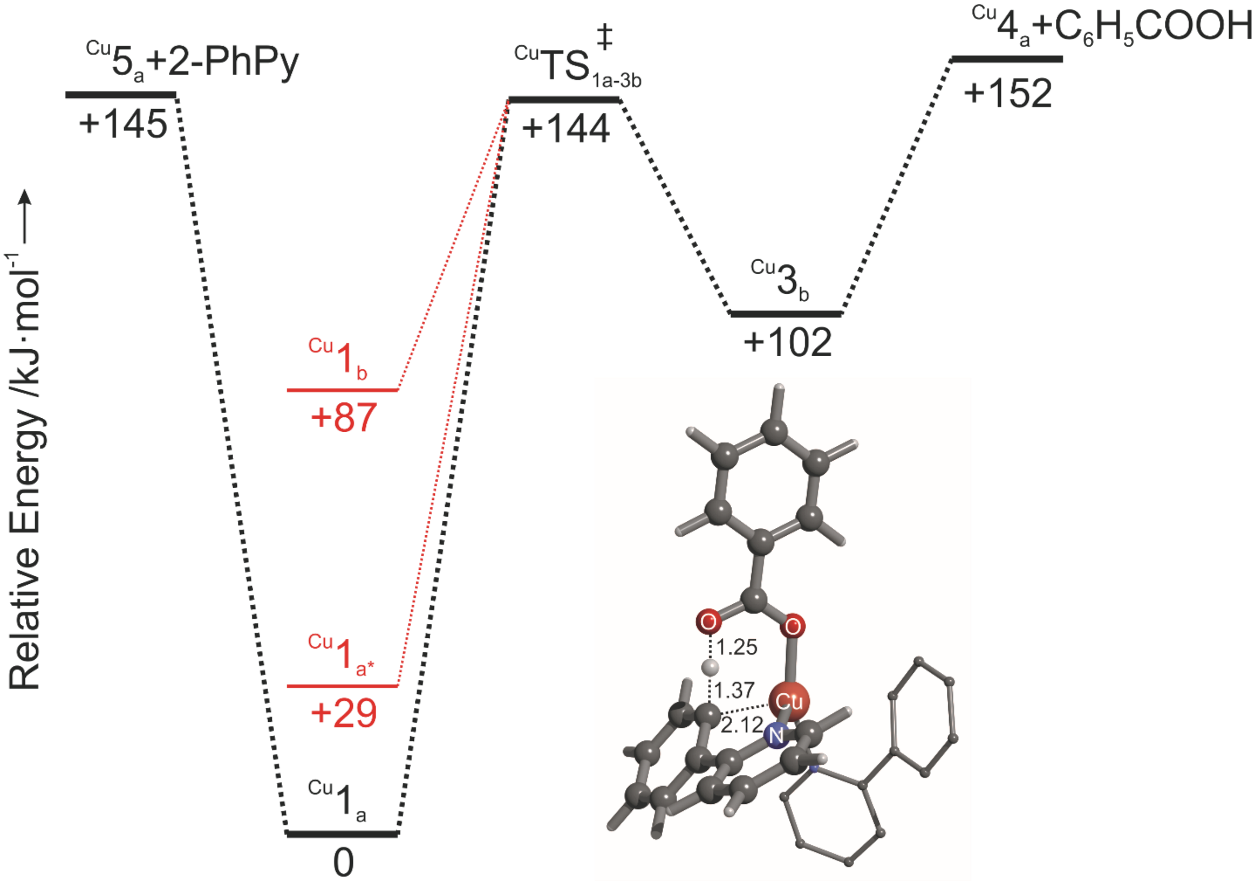

Figure S27. Zero point potential energy surface (B3LYP/6-31G*:SDD(Cu)) for the Cu assisted C-H activation of 2-PhPy with a $C_6H_5COO^-$ counter ion. Representations of structures are shown in Figure 2. All distances are in Å.

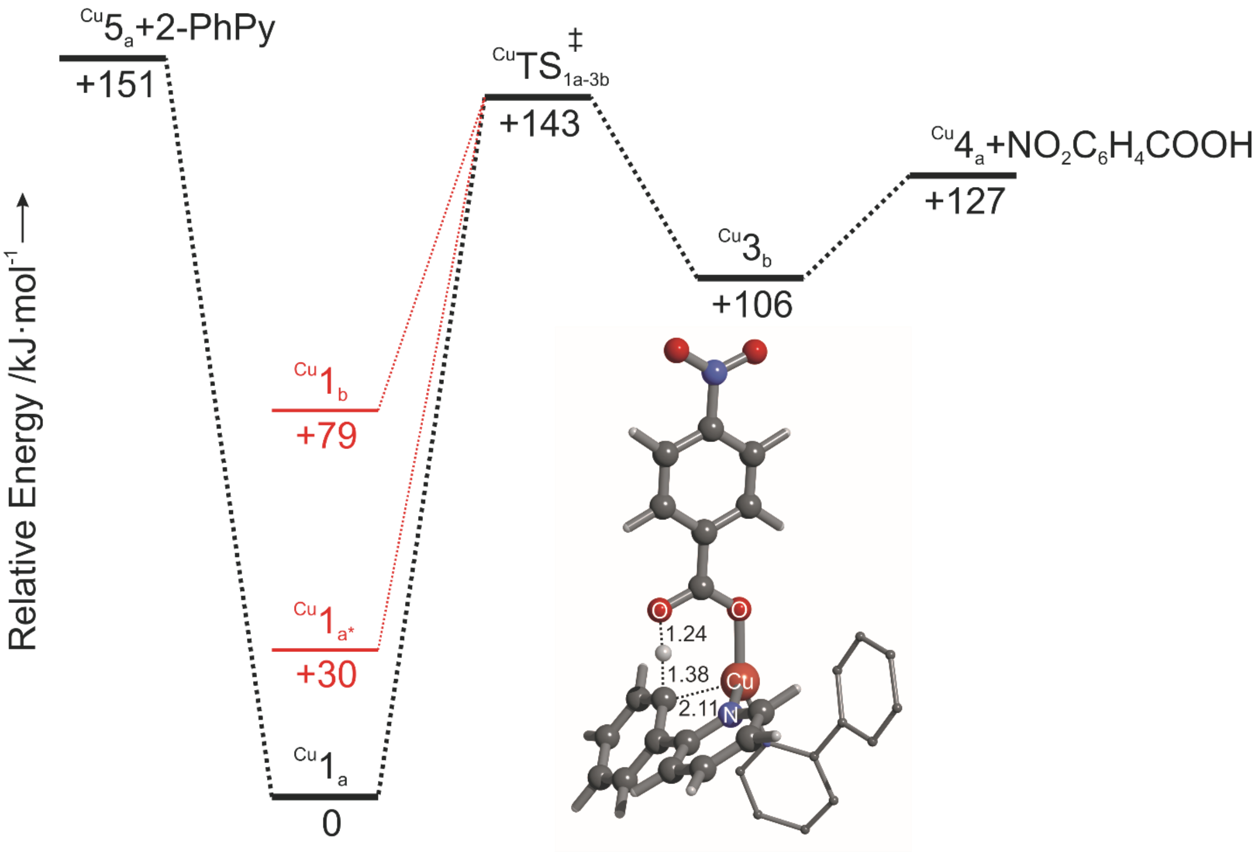

Figure S28. Zero point potential energy surface (B3LYP/6-31G*:SDD(Cu)) for the Cu assisted C-H activation of 2-PhPy with a $NO_2C_6H_4COO^-$ counter ion. Representations of structures are shown in Figure 2. All distances are in Å.

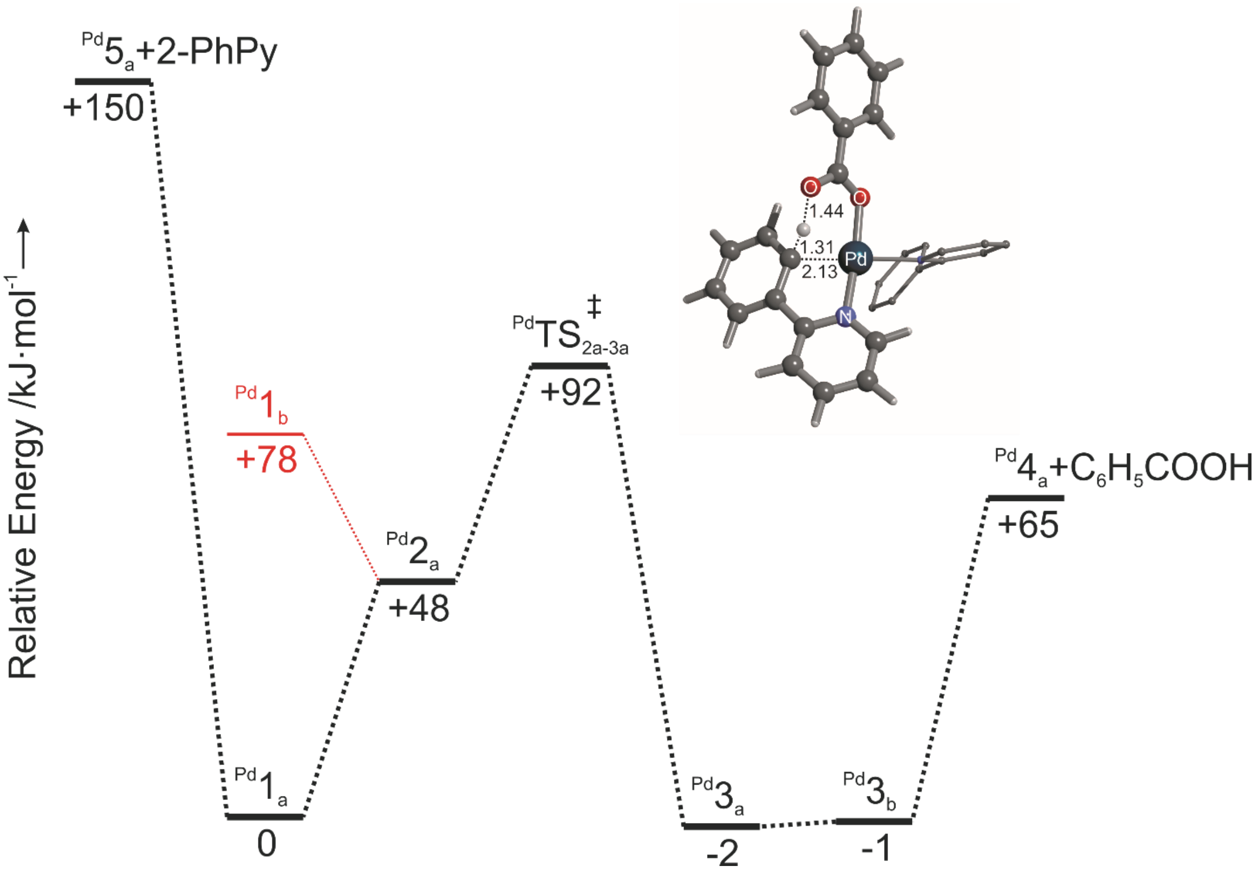

Figure S29. Zero point potential energy surface (B3LYP/6-31G*:SDD(Pd)) for the Pd assisted C-H activation of 2-PhPy with a $C_6H_5COO^-$ counter ion. Representations of structures are shown in Figure 2. All distances are in Å.

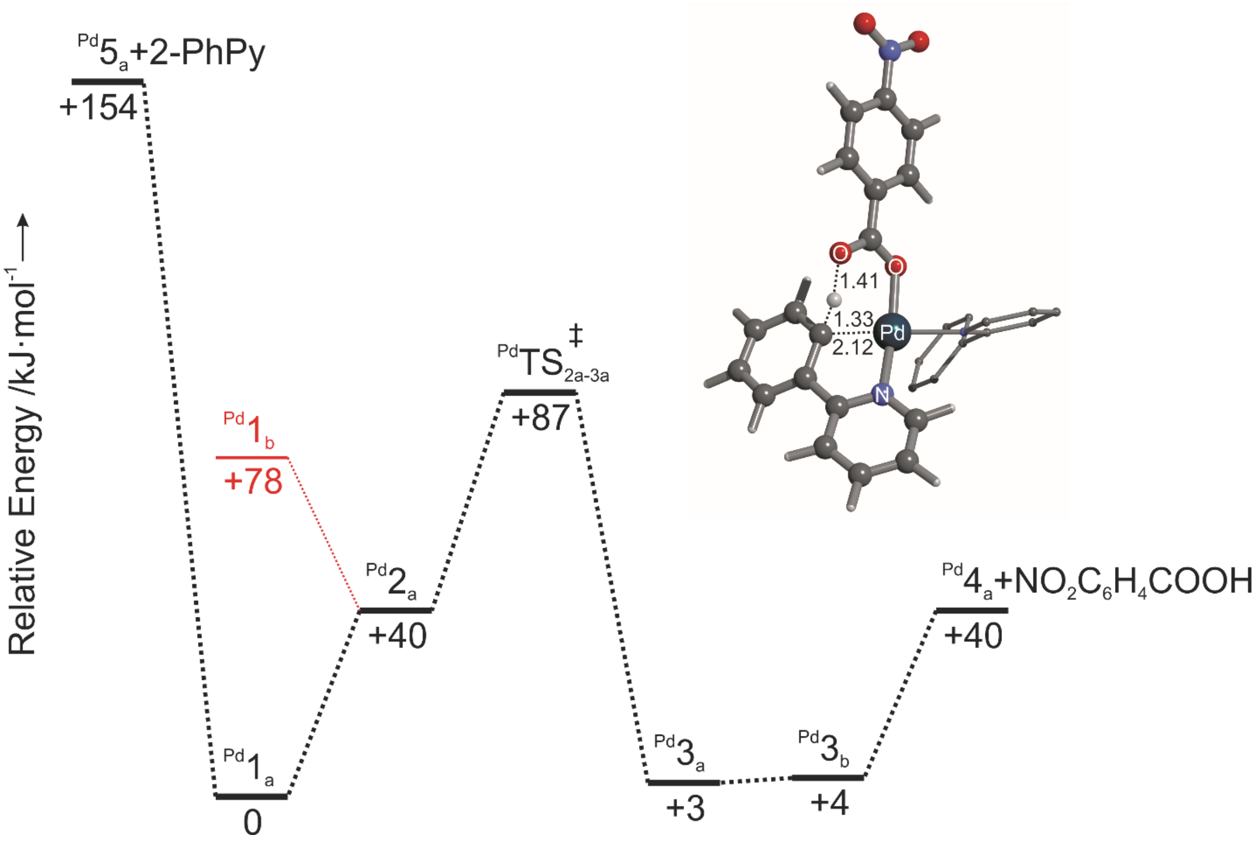

Figure S30. Zero point potential energy surface (B3LYP/6-31G*:SDD(Pd)) for the Pd assisted C-H activation of 2-PhPy with a $NO_2C_6H_4COO^-$ counter ion. Representations of structures are shown in Figure 2. All distances are in Å.

6. Trifluoroacetic acid DFT study

We have calculated corresponding TS bond lengths and potential energy surfaces with a trifluoroacetate counter ion in place of acetate for all three of the catalytic processes previously shown. Equivalent structures were located and each of the reactions appears likely to proceed in the same manner as previously observed with the acetate counter ion. Structures are thus labelled in the same format. Calculations were performed in the same fashion as with an acetate counter ion.

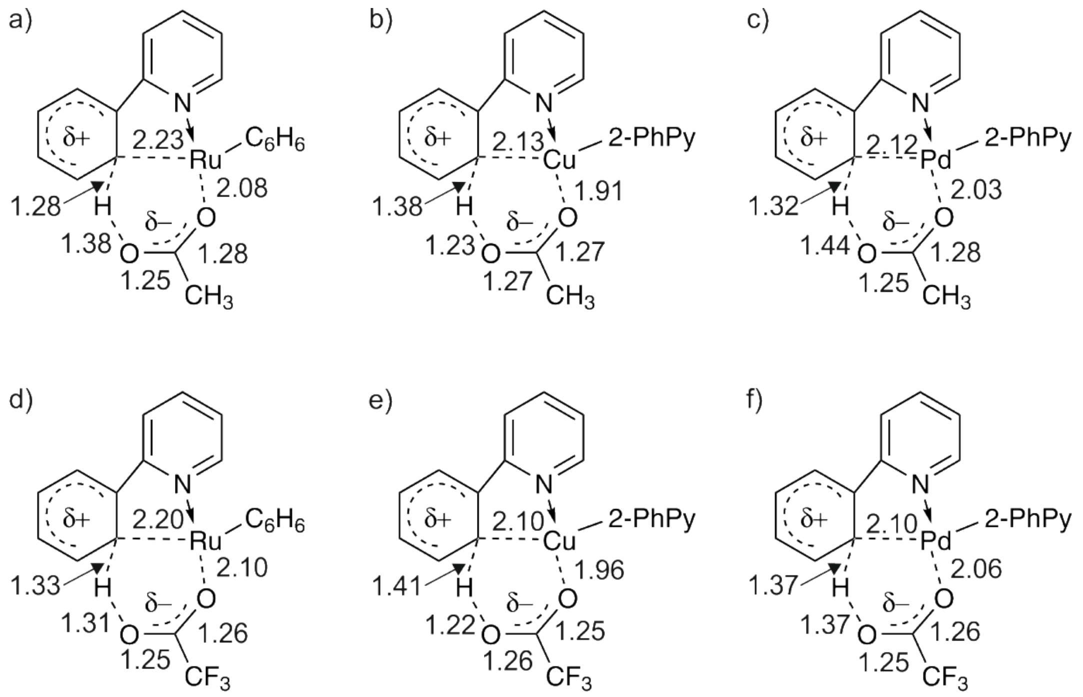

Figure S31. 6-membered transition structures showing key bond lengths for (a) $^{Ru}TS_{2a\text{-}3a}$, (b) $^{Cu}TS_{1a\text{-}3b}$ and (c) $^{Pd}TS_{2a\text{-}3a}$ with an acetate counter ion and (d) $^{Ru}TS_{2a\text{-}3a}$, (e) $^{Cu}TS_{1a\text{-}3b}$, and (f) $^{Pd}TS_{2a\text{-}3a}$ with a trifluoroacetate counter ion. All distances are in Å.

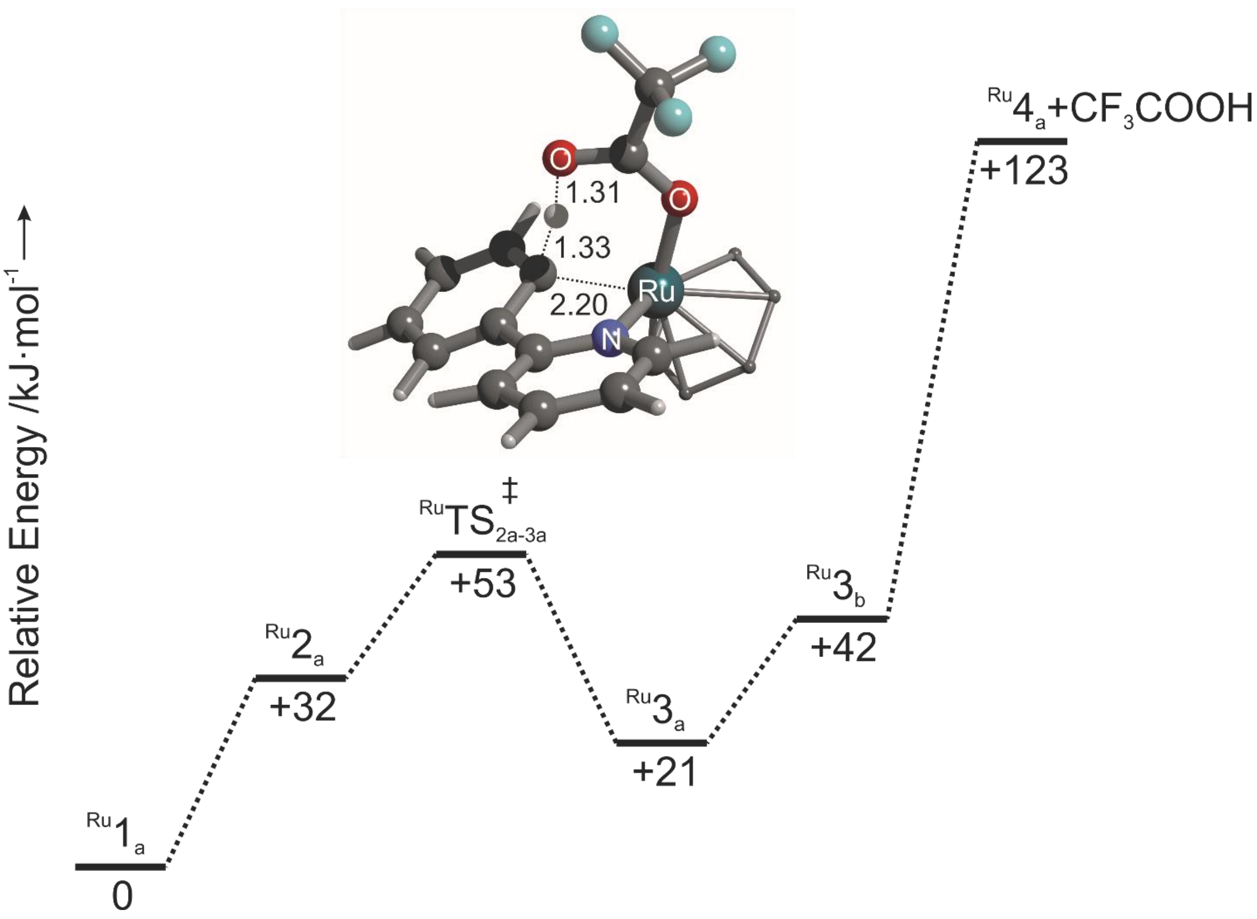

Figure S32. Zero point potential energy surface (B3LYP/cc-pVTZ:cc-pVTZ-pp(Ru)) for the Ru assisted C-H activation of 2-PhPy with a CF_3COO^- counter ion. Representations of structures are shown in Figure 2. All distances are in Å.

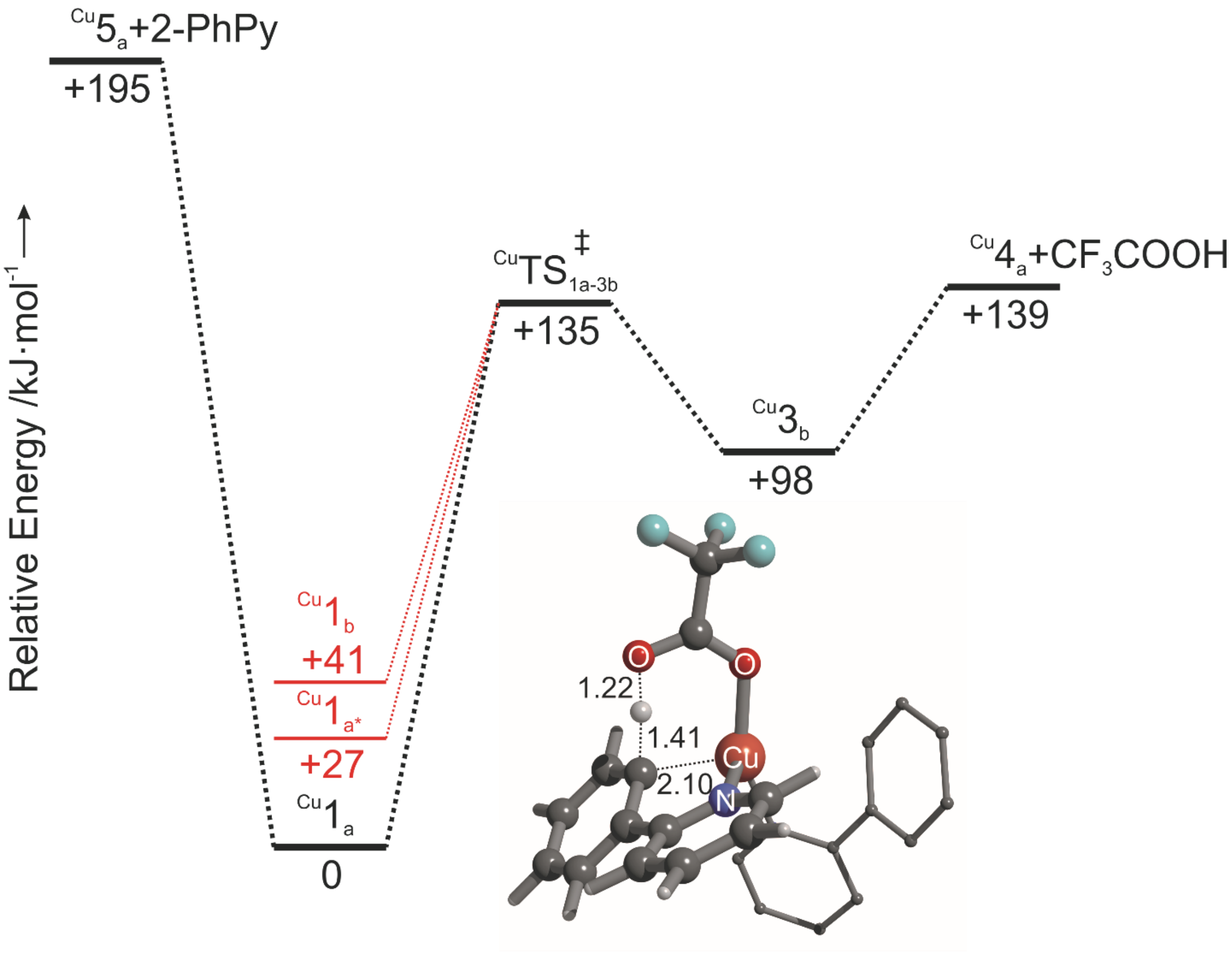

Figure S33. Zero point potential energy surface (B3LYP/cc-pVTZ:cc-pVTZ-pp(Cu)) for the Cu assisted C-H activation of 2-PhPy with a CF_3COO^- counter ion. Representations of structures are shown in Figure 2. All distances are in Å.

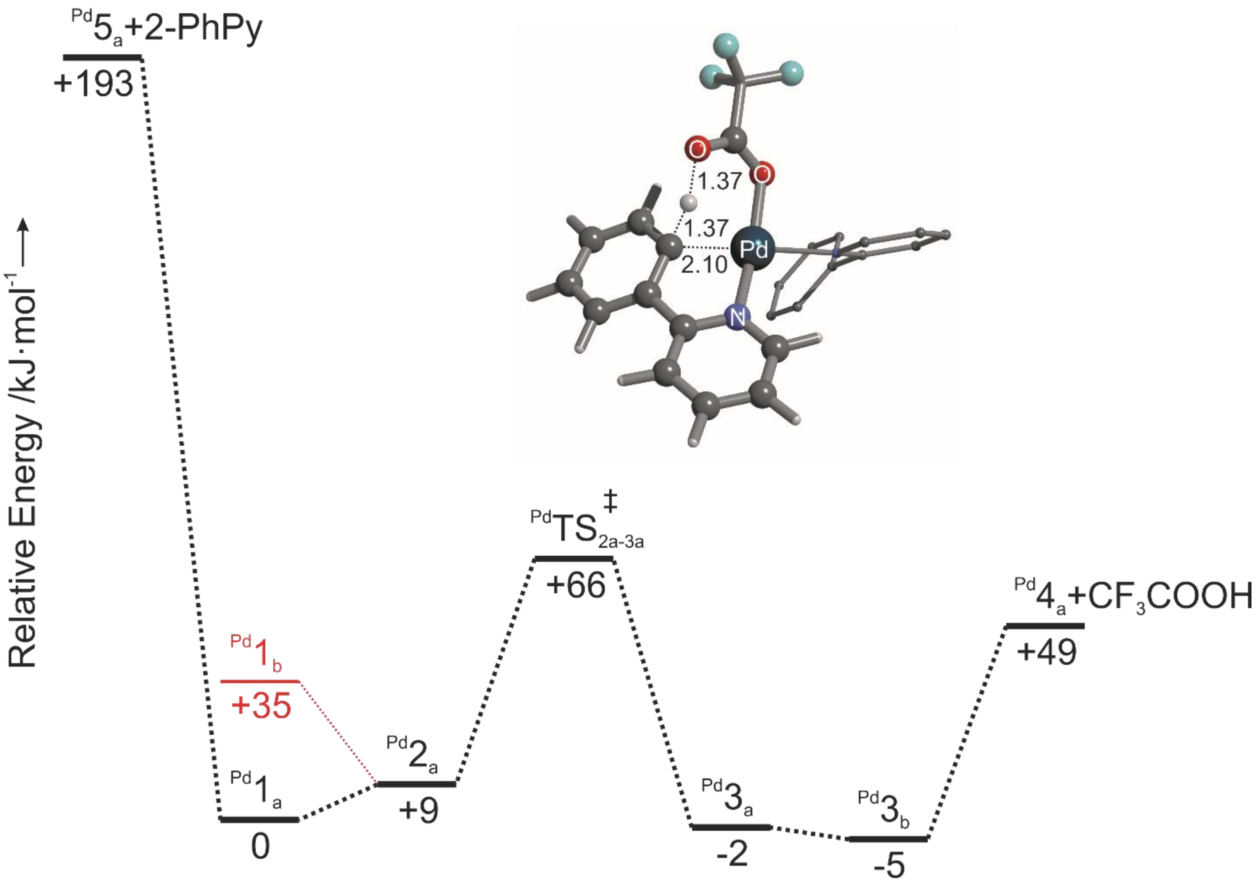

Figure S34. Zero point potential energy surface (B3LYP/cc-pVTZ:cc-pVTZ-pp(Pd)) for the Pd assisted C-H activation of 2-PhPy with a CF_3COO^- counter ion. Representations of structures are shown in Figure 2. All distances are in Å.

7. Laser power dependence

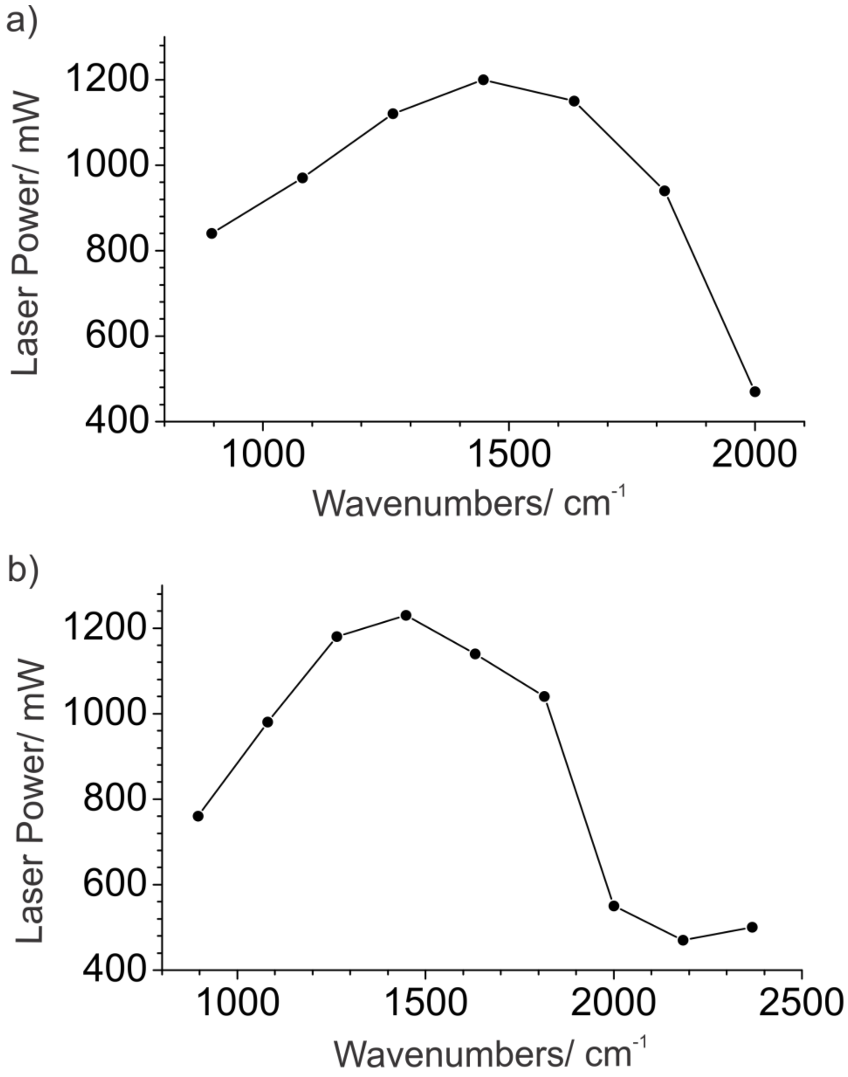

Figure S35. Dependence of the free electron laser power on the wavelength during measurement of the IRMPD spectra reported in the main article shown in a) Figure 10a; b) Figure 11a and Figure 12a.

References:

(1) Lioe, H.; O'Hair, R. a J. *Org. Biomol. Chem.* **2005**, *3*, 3618–3628.

(2) Vikse, K. L.; Henderson, M. a; Oliver, A. G.; McIndoe, J. S. *Chem. Commun. (Camb).* **2010**, *46*, 7412–7414.
